# Exploring inflammation‐related protein expression and its relationship with TSPO PET in Alzheimer's disease

**DOI:** 10.1002/alz.70171

**Published:** 2025-04-28

**Authors:** Ilaria Pola, Nicholas J. Ashton, Marco Antônio De Bastiani, Wagner S. Brum, Nesrine Rahmouni, Kubra Tan, Luiza Santos Machado, Stijn Servaes, Jenna Stevenson, Cécile Tissot, Joseph Therriault, Tharick A. Pascoal, Kaj Blennow, Henrik Zetterberg, Eduardo R. Zimmer, Pedro Rosa‐Neto, Andréa L. Benedet

**Affiliations:** ^1^ Department of Psychiatry and Neurochemistry Institute of Neuroscience and Physiology The Sahlgrenska Academy University of Gothenburg Mölndal Sweden; ^2^ Banner Alzheimer's Institute and University of Arizona Phoenix Arizona USA; ^3^ Centre for Age‐Related Medicine Stavanger University Hospital Stavanger Norway; ^4^ King's College London Institute of Psychiatry Psychology & Neuroscience Maurice Wohl Clinical Neuroscience Institute London UK; ^5^ NIHR Biomedical Research Centre for Mental Health and Biomedical Research Unit for Dementia at South London and Maudsley NHS Foundation London UK; ^6^ Graduate Program in Biological Sciences: Biochemistry Universidade Federal do Rio Grande do Sul (UFRGS) Porto Alegre Brazil; ^7^ McGill Centre for Studies in Aging McGill University Verdun Quebec Canada; ^8^ Department of Neurology and Neurosurgery Faculty of Medicine McGill University Verdun Quebec Canada; ^9^ Lawrence Berkeley National Laboratory Berkeley California USA; ^10^ Departments of Psychiatry University of Pittsburgh School of Medicine Pittsburgh Pennsylvania USA; ^11^ Department of Neurology School of Medicine University of Pittsburgh Pittsburgh Pennsylvania USA; ^12^ Clinical Neurochemistry Laboratory Sahlgrenska University Hospital Mölndal Sweden; ^13^ Department of Neurodegenerative Disease UCL Institute of Neurology, Queen Square London UK; ^14^ UK Dementia Research Institute at UCL London UK; ^15^ Hong Kong Center for Neurodegenerative Diseases, Clear Water Bay Hong Kong China; ^16^ Wisconsin Alzheimer's Disease Research Center University of Wisconsin School of Medicine and Public Health University of Wisconsin‐Madison Madison Wisconsin USA; ^17^ Department of Pharmacology Graduate Program in Biological Sciences: Pharmacology and Therapeutics Universidade Federal do Rio Grande do Sul (UFRGS) Porto Alegre Brazil; ^18^ Brain Institute of Rio Grande do Sul PUCRS Porto Alegre Brazil

**Keywords:** Alzheimer's disease, biomarkers, cerebrospinal fluid, neuroinflammation, TSPO PET

## Abstract

**INTRODUCTION:**

To understand the role of neuroinflammation in Alzheimer's disease (AD), we characterized immune‐related proteins in central and peripheral biofluids.

**METHODS:**

Selection of participants from the Translational Biomarker of Aging and Dementia (TRIAD) cohort with available translocator protein (TSPO) positron emission tomography (PET), cerebrospinal fluid (CSF) (*n* = 97), and plasma (*n* = 165). Biofluid samples analyzed with Olink technology (368 inflammation proteins).

**RESULTS:**

Elevated proteins levels in CSF of TSPO‐positive individuals were identified. Functional enrichment analysis of CSF proteins revealed processes implicated in AD (MAPK, ERK cascades, cytokine, and leukocyte signaling). Selected candidates (CXCL1 and TNFRSF11B) showed high correlation with each other in CSF and with TSPO PET signal, but weaker associations with amyloid and tau PET. No significantly changed proteins in plasma between TSPO groups were found.

**DISCUSSION:**

This explorative study identified two potential targets in CSF showing correlations with TSPO, amyloid and tau PET, suggesting a direct link between neuroinflammation, expression of these proteins and their potential implication in AD.

**Highlights:**

- Several proteins are elevated in CSF of TSPO PET‐positive individuals, linking them to neuroinflammation.
- Elevated CSF proteins were enriched in pathways such as MAPK, ERK, and cytokine signaling, linking them to the AD pathophysiology.
- Candidate proteins (CXCL1 and TNFRSF11B) correlated strongly with TSPO PET, particularly in brain regions known to be affected in AD.
- Although none of the plasma proteins remained significant after multiple comparisons correction when comparing their expression between TSPO groups, as done for CSF, candidate CSF proteins were found to correlate with plasmatic proteins, highlighting the complexity of the immune system.

## 1 BACKGROUND

Alzheimer's disease (AD) is a progressive pathophysiological process characterized by the accumulation of amyloid‐*β* (Aβ) and tau proteins, ultimately leading to neurodegeneration and cognitive decline.^1^, ^2^ In this context, neuroinflammation has been suggested as a significant contributor to AD progression.^3^, ^4^ Studies in mouse models of Aβ and tau suggest that microglial reactivity may accelerate Aβ plaque deposition and tau spreading, with immune‐related alterations observed in both brain tissue and peripheral biofluids.^5^, ^6^

Despite extensive research, the precise role of the immune system in AD remains unclear, with conflicting views on its beneficial impact ^7^, ^8^, ^9^ or detrimental effect.^10^, ^11^, ^12^ Imaging techniques, such as positron emission tomography (PET) using translocator protein (TSPO) radiotracers, have provided valuable insights into neuroinflammation, showing increased signal in amyloid PET‐positive mild cognitive impairment (MCI) patients compared with amyloid PET‐negative individuals.^13^ Of note, TSPO PET imaging has been shown to be increased in neurodegenerative diseases and central inflammatory disorders, such as multiple sclerosis,^14^, ^15^ and in AD, it was shown to reflect disease stages by indicating different microglial phenotypes associated with neurodegeneration.^16^ Particularly, while at early AD stages (Braak stages 0‐II), TSPO‐expressing microglia may reflect a phagocytic phenotype, marked by CD68, implicating a potential protective role, at later stages (Braak V‐VI), TSPO‐expressing microglia are more associated with a reactive, scavenging phenotype, marked by MDR‐A+, associating it to neurodegeneration and worsening of cognitive decline. Several tracers have been explored in the recent research. Particularly, [^11^C]PBR28, a second‐generation TSPO radioligand, demonstrated high affinity for the TSPO binding site expressed on activated microglia and astrocytes. Compared to its predecessor [¹¹C]‐PK11195, [^11^C]PBR28 offers a significantly improved signal‐to‐noise ratio, enhancing the applicability in neuroinflammation imaging.^17^

While AD core fluid biomarkers, including Aβ, phosphorylated tau, along with additional markers such as total tau and neurofilament light, have provided valuable insights, conflicting findings for candidate inflammation biomarkers have been reported. Beyond variability in cohort characteristics and immune system dynamic changes, assay sensitivity and pre‐analytical handling may play a critical role on these inconsistencies. However, novel technological advancements have substantially improved the sensitivity of biochemical assays, leading to more consistent findings across diverse cohorts worldwide.

Cerebrospinal fluid (CSF) and plasma biomarkers related glial function, such as soluble triggering receptor expressed on myeloid cells 2 (sTREM2), chitinase‐3‐like protein 1 (YKL‐40) and glial fibrillary acidic protein (GFAP) have shown promise in reflecting neuroinflammation levels across different disease stages and correlating with core AD biomarkers.^18^, ^19^, ^20^, ^21^, ^22^, ^23^ Although most expressed in microglial and astrocytes, whether these markers reflect glial‐related neuroinflammation still needs properly validation. No neuropathological validation exists for sTREM2 and YKL‐40 and blood GFAP and showed weak correlation with GFAP in *post mortem* tissue.^10^, ^24^ In fact, developing neuroinflammation fluid biomarkers is challenging because these glial cells undergo constant morphological, functional and molecular changes.^25^, ^26^ For blood biomarkers, it is even more complex given its greater distance to the brain, compared with CSF. Blood GFAP performance is an exception showing good performance,^20^ whereas other inflammation‐related biomarkers with clear changes in the CSF of AD patients, such as sTREM2 and YKL‐40, have shown mixed results in plasma.^27^, ^28^, ^29^, ^30^

Overall, previous research, has given much effort in linking peripheral biofluid markers in blood and CSF to central neuroinflammation, suggesting a peripheral‐central inflammatory axis. Studies have shown that elevated cytokines and chemokines, such as IL‐6, CCL2, and CCL11, are present in both CSF and plasma and correlate with microglial activation as measured by TSPO PET,^31^, ^32^ Furthermore, elevated inflammatory markers like interleukin (IL)‐1β, tumor necrosis factor α (TNF‐α), and IL‐6 have been reported in *post mortem* AD brain specimens, correlating with neurodegeneration, highlighting their association with microglial activation and neuroinflammation.^33^ Indeed, *post mortem* study on human brains found correlations between the binding of tracers (11C‐PK11195 and 11C‐DAA1106) and activated microglia and not with reactive astrocytes in various central nervous system (CNS) diseases.^34^

 Although there is an interconnection between peripheral inflammation and central processes, further research is required to address this challenge.

To date, no blood biomarker for AD, or other brain disorders, has been directly discovered in plasma via proteomics without previously being verified in the CSF. Therefore, the importance of biomarker discovery efforts leveraging on both CSF and blood data cannot be overstated in AD.

This explorative study aims at identifying potential CSF and blood biomarkers of neuroinflammation in relation to TSPO PET signal across the AD spectrum. To enhance the relevance of findings to AD, participants were selected based on AD‐specific biomarkers (amyloid and tau PET), and proteins were analyzed in the context of established AD‐related pathways. Such preliminary findings could provide novel insights about disease pathophysiology, determining when, which, and how immune responses are affecting the AD cascades, paving the way for future research with larger, more representative samples.

**RESEARCH IN CONTEXT**

1. **Systematic review**: With this study we aimed at identifying potential immune‐related proteins involved in the Alzheimer's disease (AD) pathology and closely associated with neuroinflammation. We analyzed data from 165 patients in the AD continuum with available imaging data on neuroinflammation (proxied by translocator protein (TSPO) positron emission tomography [PET]) together with proteomic data on plasma and cerebrospinal fluid (CSF), using Olink technology targeting 368 inflammation‐related proteins.
2. **Interpretation**: We identified several proteins that were elevated in the CSF of TSPO‐positive individuals compared with TSPO‐negative individuals, showing a stepwise increase from lower to higher TSPO PET binding. Our study provides evidence linking neuroinflammation with specific CSF inflammatory proteins in AD, with high inter‐protein correlations, combined with significant associations across brain regions, underscoring the complex network of interactions driving AD pathology. The scenario in plasma revealed to be more complex, supporting the intricacy of immune responses in the body brain interactions.
3. **Future directions**: Our study reveals how multiplexed assays could be implemented in the investigation of novel inflammation biomarkers and points out to two potential CSF biomarkers of neuroinflammation. To further expand our findings, larger and other deeply phenotyped cohorts should be used, and more sensitive technologies should be employed for the detection of the proteins, especially in plasma.

## 2 METHODS

### 2.1 Study population

For this preliminary study, participants from the Translational Biomarker of Aging and Dementia (TRIAD) cohort were selected with available TSPO PET data. The TRIAD study was launched in 2017 as part of the McGill Centre for Studies in Aging (Montreal, Canada), with the aim of describing biomarker trajectories and interactions as drivers of dementia. The participants were genotyped for the *TSPO* gene's Ala147Thr polymorphism (rs6971), which predicts the binding affinity of [^11^C]PBR28 to the TSPO protein. To ensure comparable results, only high‐affinity binders were scanned. At the time of data analysis, the cohort included 174 individuals that underwent TSPO PET imaging, of which 165 had plasma samples available, and 97 had both CSF and plasma samples. Biofluid samples were analyzed using a Proximity Extension Assay (PEA) technology targeting 368 inflammation‐related proteins (Olink, Sweden). In addition to clinical diagnosis, the participants were here also categorized according to amyloid PET ([^18^F]AZD4694) as Aβ+ or Aβ−, with Aβ positivity defined based on a neocortical standardized uptake ratio (SUVR) of > 1.55.^35^ We included cognitively unimpaired (CU) participants, which comprised middle‐aged adults (age range, 50–86 years) (CSF *n* = 38, plasma *n* = 21) and young participants (age range, 20–30 years) (CSF *n* = 19, plasma *n* = 84) who had no objectively defined cognitive impairment (Clinical Dementia Rating [CDR] = 0). Included MCI participants (CSF *n* = 22, plasma *n* = 33) had preserved daily living activities but presented with subjective and objective (delayed recall performance on the Wechsler Memory scale) cognitive impairment, with CDR score of 0.5. AD patients (CSF *n* = 10, plasma *n* = 18) underwent clinical assessment, did not present clinical evidence of other neurological conditions (cerebrovascular or co‐existing inflammatory disease) and met the National Institute of Aging on the Alzheimer's Association criteria for probable AD determined by a physician, with a CDR score greater than 1. The non‐AD dementia (CSF *n* = 8, plasma *n* = 9) comprised individuals negative for amyloid pathology based on PET imaging and clinically diagnosed as cerebral amyloid angiopathy (CAA; *n* = 1), progressive supranuclear palsy (PSP, *n* = 2), and frontotemporal dementia (FTD; *n* = 5). The atypical dementia population included individuals who did not meet the criteria for AD and clinical diagnosis was made based on determined by the clinician based on appropriate clinical guidelines for each disorder.^36^, ^37^ The exclusion criteria comprised of inability to speak English or French; deficient auditory and visual capacities for neuropsychological assessment; major surgery; recent head trauma; active substance abuse; medical contraindication for magnetic resonance imaging (MRI) or PET; concurrently enrollment in other studies; neurological, untreated, or inadequately managed psychiatric or systemic comorbidities.^38^

All participants provided written informed consent, and ethical approval was obtained by the Douglas Mental Health University Institute Research Ethics Board (MP‐18‐2017‐157) and the Montreal Neurological Institute (MNI) PET working committee.

### 2.2 Immune‐related proteins quantification

CSF (*n* = 97) and plasma (*n* = 165) samples from a subset of individuals with available TSPO PET were analyzed by the Olink Explore 368 Inflammation proteomic panel, employing PEA technology for measurement. Protein concentrations for 368 proteins were quantified as Normalized Protein Expression (NPX) values, an arbitrary unit on a Log2 scale derived from normalized cycle threshold (Ct) values. To ensure quality control (QC), NPX values for each protein were scaled, with points falling above quantile 2 or below quantile ‐2 being excluded. The full list of the 368 proteins and validation data for the assay is accessible on the Olink website (www.olink.com).

### 2.3 PET acquisition and processing

All PET images were acquired using a Siemens High Resolution Research Tomograph (Siemens Medical Solutions, Knoxville, TN). PET images were corrected for attenuation, motion, dead time, decay, and scattered and random coincidences. PET images were automatically aligned with the native T1‐weighted MRI using linear transformations. The PET images were subsequently registered to the MNI space by applying the transformations from the PET to native MRI and from the native MRI to the template space. PET images were smoothed to achieve a final resolution of 8‐mm full width at half maximum. Amyloid PET ([^18^F]AZD4694) images were acquired at 40–70 min post‐injection of the tracer and a global SUVR was derived from average retention in the precuneus, the cingulate, inferior parietal, medial prefrontal, lateral temporal, and orbitofrontal cortices. Cerebellar grey matter was used as reference region. Aβ status was assessed using [18F]AZD4694 PET SUVR threshold of > 1.55.^35^ Tau PET ([^18^F]MK6240) images were acquired at 90–110 min post‐injection of the tracer and cerebellar gray matter was used as reference region. Global tau PET load was estimated from the average tracer retention on the temporal meta‐region of interest (ROI) (including the entorhinal, amygdala, fusiform, inferior and middle temporal cortices), which threshold for positivity has been previously defined as > 1.24 SUVR.^39^ TSPO PET ([^11^C]PBR28) images were acquired at 60 to 90 min post‐injection of the tracer and SUVR was calculated using the whole cerebellum gray matter as reference region. In our analyses, the regions used to calculate the TSPO PET SUVR were determined based on the voxel‐wise statistical differences in in average [11C]PBR28 binding between CU amyloid‐negative group (*n* = 85) and the AD dementia group (*n* = 18). The resulting parametric map was then corrected for multiple comparisons using random field theory (REF), and the brain regions that remained significant (see Figure S1) were then used for the generation of the composite mask and ROI SUVR extraction. [^11^C]PBR28 PET positivity was assigned when global [^11^C]PBR SUVR exceeded 2.5 standard deviations (2.5 SD) from the average of young participants. This threshold was set to capture abnormal [^11^C]PBR28 binding, indicating the presence of neuroinflammation within the brain.^32^

For all three PET modalities, SUVR values were also extracted from 45 anatomically defined brain regions, selected using the using the ANIMAL atlas.^40^ These SUVR values were used for the correlation analyses presented in the chord‐diagrams, for the visual colocalization in the brain regions, and calculation of proportion for each imaging modality (scatterplot and barplot), as described below.

### 2.4 Statistical analysis

We conducted our statistical analyses using the R Statistical Software Package (version 4.2.2). The NPX values were scaled and trimmed using threshold of ± 2 quantiles, and the remaining values were normalized using the RankNorm function. Due to the stringent selection criteria for participants undergoing TSPO PET imaging, the sample size is limited allowing an explorative study in nature. To overcome these limitations that may affect the generalizability of the results, we employed robust statistical methods for small samples sizes, namely, Linear Models for Microarray and RNA‐Seq Data (LIMMA model), well‐suited in this context due to its empirical Bayes (EB) moderation of variance estimates. LIMMA model used linear modeling (lmFit) to assess differential protein expression (DEP) across conditions ([^11^C]PBR PET positivity), adjusting for age and brain amyloid PET load. EB estimation (eBayes) was used to regularize variances and enhance statistical inference. Significant DEPs were identified using false discovery rate (FDR) adjustment and selected based on the output from the LIMMA analysis, which ranks associations according to adjusted *p*‐values from the smallest to the largest. We then conducted functional enrichment analysis (FEA) of gene ontology (GO) biological processes (BP) using the clusterProfiler package (version 4.6.2).^41^, ^42^ Using the GOSemSim package (v2.18.1),^43^, ^44^ we clustered the GO BP terms by semantic similarity and represented the similarity matrices as GO networks. For visualization of the networks, we employed the RedeR package (version 2.2.1).^45^ From the LIMMA analysis, we next selected proteins 10 proteins for further investigation, by including proteins that survived FDR correction and five extra proteins (based on their *p*‐value), allowing some flexibility, to address the reduced statistical power. To evaluate the expression of these proteins of interest across different cell types, we analyzed RNA sequencing (RNA‐seq) data from the middle temporal gyrus (MTG), obtained from the Allen Brain Atlas consortium (Human MTG 10x SEA‐AD 2022, https://portal.brain‐map.org/atlases‐and‐data/rnaseq/human‐mtg‐10x_sea‐ad). This dataset includes single‐nucleus RNA profiles from 166,868 nuclei, derived from five *post mortem* human brain samples. We processed this data to calculate the average RNA expression levels across cell types. Using annotations for cell classes and subclasses from the Allen Brain Atlas data, we derived the average RNA expression values for non‐neuronal cells (including astrocytes, endothelial, microglia, oligodendrocytes, and oligodendrocyte precursor cells [OPCs]) as well as for neuronal cells (gamma‐aminobutyric acid [GABA]ergic and glutamatergic neurons). Additionally, we calculated the proportion of RNA expression for each cell type, based on these average expression levels. To assess protein interactions, we performed Spearman correlations between the top 10 proteins. Such correlations were included in a sunburst chart, together with the correlation of such proteins with each PET imaging modality. To compare the strength of association between the proteins and the imaging modalities, we calculated Pearson correlation coefficients between each protein and the global PET SUVR measures for each of the tracers. To determine whether the correlations with TSPO PET were statistically different from those with amyloid PET and tau PET, we performed Fisher's *Z*‐tests for comparing independent correlation coefficients using the cocor package in R. To assess the relationship between the proteins and TSPO PET while controlling for the potential confounding effects of amyloid PET and tau PET, we conducted partial correlation analyses. The partial correlation model evaluated CSF proteins independently and included global SUVR quantifications for all PET modalities simultaneously. This analysis was performed using the pcor function from the ppcor package in R. Next, participants were segregated into global TSPO PET quartile groups, to replicate our initial model while adding granularity, and the CSF protein distribution was visualized with boxplots. More detailed analyses were performed to retain final proteins of interest. The top 10 proteins were further analyzed as the dependent variable and TSPO PET as the primary predictor (both as continuous variables and quartile‐based). Models were performed both adjusted for age, sex, and amyloid PET and unadjusted. This led to the selection of two final proteins. The top 2 protein levels were then correlated with the TSPO PET values in 45 anatomically‐defined brain regions, selected using the using the ANIMAL^40^ atlas in a Chord Diagram. To assess visual colocalization of the proteins, we used the brain network visualization tool Brain Net Viewer in MATLAB. In addition to the uncorrected analysis, we accounted for multiple comparisons in the correlations analysis by applying the Benjamini‐Hochberg procedure to control the FDR. Specifically, within each modality, we performed 45 tests and adjusted the *p*‐values accordingly.

## 3 RESULTS

### 3.1 Cohort characteristics

For the CSF analysis (Table 1), we assessed 97 individuals with TSPO PET (mean [SD] age, 61 [± 20] years; 59 females [60.8%], 38 males [39.2%]; years of education, 15 [± 3.1]). This is included 57 cognitively unimpaired individuals (CU, mean [SD] age, 55 [± 23] years; 40 females [70.2%], 17 males [29.8%]; years of education, 15 [± 3.2]) and 40 cognitively impaired (CI, mean [SD] age, 69 [± 7.3] years; 19 females [47.5%], 21 males [52.5%]; years of education, 15 [± 2.9]). Mean (SD) 1.7 (± 0.57) for amyloid PET SUVR, mean (SD) 1.1 (± 0.53) for tau PET SUVR, and mean (SD) 1.1 (± 0.13) for TSPO PET SUVR. Thirty‐five participants were amyloid‐positive, and 62 participants were amyloid‐negative.

**TABLE 1:** Demographics of the TRIAD participants with CSF measurements included in this study

Characteristics	Young (*N* = 19)	CU− (*N* = 28)	CU+ (*N* = 10)	MCI− (*N* = 7)	MCI+ (*N* = 15)	AD+ (*N* = 10)	Non‐AD (*N* = 8)
Age, years	23 (± 2.1)	71 (± 6.6)	72 (± 4.0)	73 (± 6.7)	72 (± 4.9)	65 (± 9.2)	65 (± 4.3)
Female, *n* (%)	10 (52.6%)	22 (78.6%)	8 (80.0%)	2 (28.6%)	7 (46.7%)	5 (50.0%)	5 (62.5%)
Years of Education	17 (± 1.6)	15 (± 3.7)	14 (± 2.9)	15 (± 4.4)	16 (± 2.6)	15 (± 2.1)	14 (± 3.2)
Aβ‐PET SUVR	1.2 (± 0.07)	1.3 (± 0.09)	2.2 (± 0.44)	1.3 (± 0.17)	2.4 (± 0.38)	2.4 (± 0.41)	1.2 (± 0.13)
Tau‐PET (SUVR)	0.82 (± 0.08)	0.83 (± 0.08)	0.92 (± 0.09)	0.84 (± 0.11)	1.3 (± 0.42)	2.1 (± 0.90)	0.81 (± 0.09)
TSPO‐PET (SUVR)	0.98 (± 0.04)	1.1 (± 0.07)	1.1 (± 0.14)	1.2 (± 0.15)	1.2 (± 0.09)	1.3 (± 0.15)	1.2 (± 0.11)
TSPO‐positive, *n* (%)	0 (0%)	15 (53.6%)	4 (40.0%)	4 (57.1%)	11 (73.3%)	10 (100%)	5 (62.5%)

Abbreviations: “+”, amyloid‐β‐positive; “−”, amyloid‐β‐negative; Aβ, amyloid‐β; AD, Alzheimer's disease; CFS, cerebrospinal fluid; CU, cognitively unimpaired; MCI, mild cognitively impaired; Non‐AD, non‐Alzheimer's disease; PET, positron emission tomography; SUVR, standard uptake value ratio; TRIAD, translational biomarker of aging and dementia.

For plasma analysis (Table S1), we assessed 165 individuals with TSPO PET (mean [SD] age, 64 [± 18] years; 109 females [66.1%)], 56 males [33.9%]). This included 105 cognitively unimpaired individuals (CU, mean [SD] age, 61 [± 21] years; 80 females [76.2%], 25 males [23.8%]) and 60 cognitively impaired (CI, mean [SD] age, 70 [± 8.2] years; 29 females [48.3%], 31 males [51.7%]). Mean (SD) 1.6 (± 0.57) for amyloid PET SUVR, mean (SD) 1.1 (± 0.57) for tau PET SUVR, and mean (SD) 1.1 (± 0.12) for TSPO PET SUVR. Fifty‐eight participants were amyloid‐positive, and 107 participants were amyloid‐negative.

Other characteristics, including amyloid status and cognitive status with Mini‐Mental State Examination (MMSE), of the TSPO PET‐positive and ‐negative groups are included in Table S2.

**FIGURE 1:**
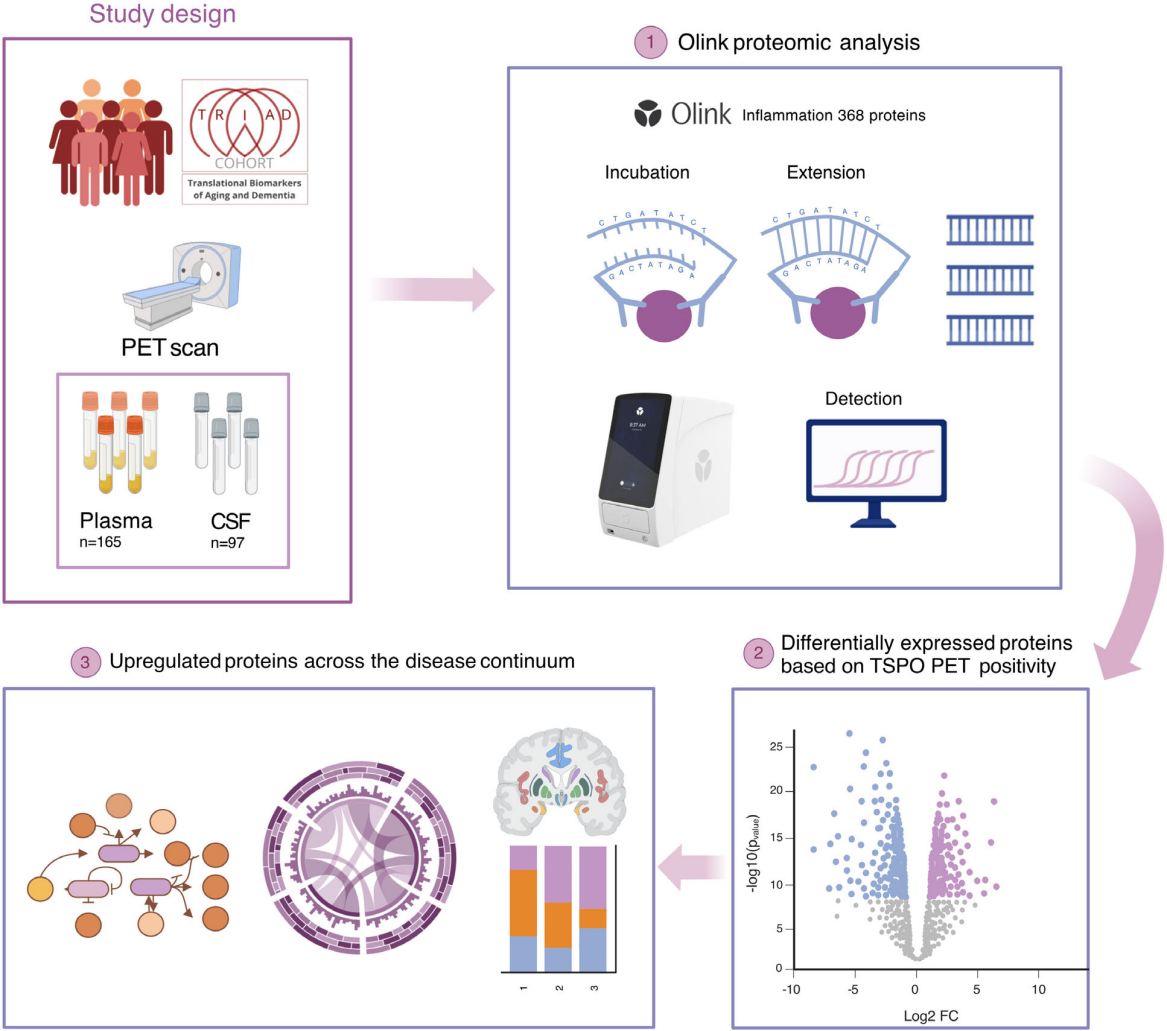
CSF (*n* = 97) and plasma (*n* = 165) samples from the TRIAD cohort, comprised of PET‐staged individuals across the AD continuum, were analyzed by the Olink Explore 368 Inflammation proteomic panel (1). Next, we assessed DEPs across TSPO PET‐positive and ‐negative participants (2) and we subsequentially performed several statistical analyses to evaluate their gene ontology and relationship with the pathways that could be linked to the disease, their cell expression, the relationship of top 10 biomarkers with amyloid PET, tau PET and TSPO PET and the relationship of these protein in AD specific brain regions (3). AD, Alzheimer's disease; CFS, cerebrospinal fluid; DEP, differential protein expression; PET, positron emission tomography; TRIAD, Translational Biomarker of Aging and Dementia; TSPO, translocator protein.

An overview of the study design and main analyses is displayed in Figure 1.[Fig alz70171-fig-0002]

### 3.2 Differentially expressed CSF proteins in TSPO PET‐positive and ‐negative individuals

**FIGURE 2 alz70171-fig-0002:**
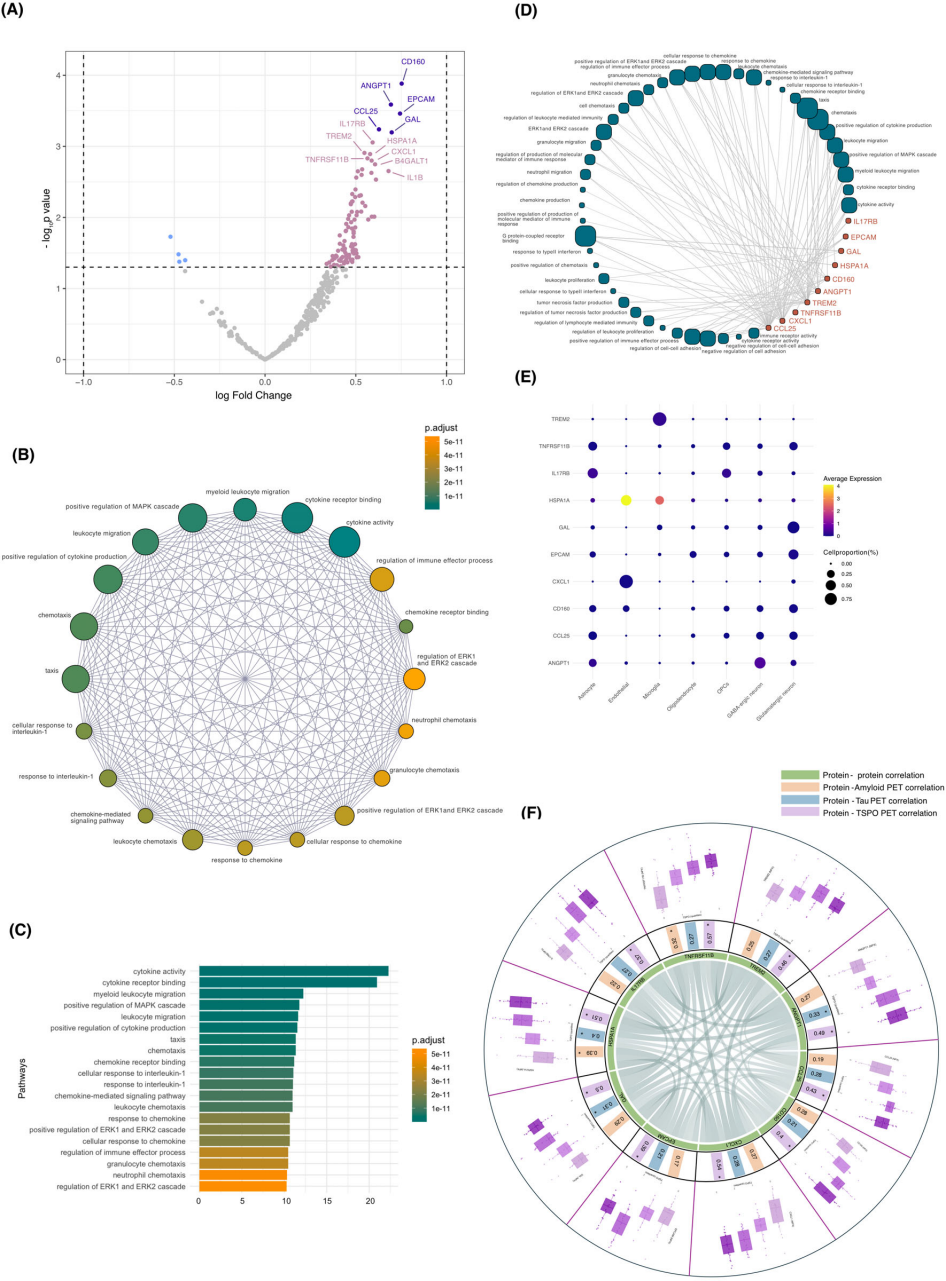
(A) Volcano plot showing the association among 368 CSF proteins in TSPO PET‐positive versus ‐negative participants by displaying the log2 fold change of protein abundance in CSF (*x*‐axis) against statistical significance (*y*‐axis) between individuals positive and negative for TSPO PET. The proteins colored in pink represent the proteins upregulated in TSPO PET‐positive participants (nominally significant, *p* < 0.05). The proteins colored in blue represent the proteins upregulated in TSPO PET‐negative participants (nominally significant, *p* < 0.05). The proteins colored in purple represent the proteins upregulated in *TSPO PET*‐positive participants with *p* < 0.05 after FDR correction. (B) Enrichment map showing GO enriched terms into a network with edges connecting overlapping gene/protein sets. (C) Barplot showing the top 20 GO terms by FEA Q‐scores. The bars represent the Q‐scores, with colors indicating the adjusted *p*‐values (gradient from dark green to yellow indicating increasing significance, with darker shades representing higher Q‐scores). The *x*‐axis shows the GO term descriptions, ordered by Q‐score, and the *y*‐axis represents the Q‐scores. (D) Enrichment map of the top 10 proteins selected for further analyses. (E) Proportion of expression by cell type from single‐cell transcriptomics data from the middle temporal gyrus for the top 10 proteins (F) Protein level changes and correlations with amyloid, tau and TSPO PET. Each segment of the circle represents one of the 10 candidate proteins. The inner layer (layer 1) represents a chord diagram of top 10 proteins from LIMMA analysis CD160, ANGPT1, EPCAM, CCL25, GAL, HSPA1A, IL17RB, TREM2, TNFRSF11B, CXCL1 and tau PET, amyloid PET and TSPO PET. The intensity of correlation is visually represented by color intensity in the circular plot (corrplot). Darker colors indicate stronger positive correlations. Only significant correlations are displayed. The middle layer (layer 2) displays the correlations each of the top 10 proteins and their correlations with amyloid PET (in orange), tau PET (in blue) and TSPO PET (in purple). All correlation values are displayed, with only values presenting a * being significant. The outer layer (layer 3) shows TSPO PET Quartile Grouping (ROI based, *x*‐axis) and distribution of top 10 significant protein levels (NPQ values, *y*‐axis) in the CSF, visualized with boxplots. CFS, cerebrospinal fluid; FDR, false discovery rate; FEA, functional enrichment analysis; GO, gene ontology; PET, positron emission tomography; ROI, region of interest; TSPO, translocator protein.

The LIMMA analysis unveiled 22 DEPs when contrasting TSPO PET‐positive and ‐negative individuals (Figure 2A), nominal and adjusted values are presented in Table S3. DEPs included CD160 (*P*_adj_ = 0.042, *B*‐statistic = 0.910, logFC = 0.753), Angiopoietin 1 (ANGPT1; *P*_adj_ = 0.042, *B*‐statistic = 0.336, logFC = 0.693), epithelial cell adhesion molecule (EPCAM; *P*_adj_ = 0.042, *B*‐statistic = 0.079, logFC = 0.743), C‐C motif chemokine ligand 25 (CCL25; *P*_adj_ = 0.047, *B*‐statistic = −0.360, logFC = 0.628), and Galanin (GAL; *P*_adj_ = 0.047, B‐statistic = −0.443, logFC = 0.698). The volcano plot highlighted proteins that are more expressed in the CSF of participants with high TSPO PET binding compared to those with low TSPO PET binding.

### 3.3 Heterogeneity of biological pathways as a function of TSPO PET‐positivity

To characterize the BP and pathways that were significantly associated with TSPO PET binding, we performed enrichment analysis on DEPs as a function of TSPO PET positivity (LIMMA model, with a *p* < 0.01), unveiling BP that have been repeatedly reported as being associated to AD, such as *positive regulation of MAPK cascade* (*P*_adj_ = < 0.0001), *positive regulation of ERK1 and ERK2 cascade* (*P*_adj_ = < 0.0001), *cytokine‐mediated signaling pathway* (*P*_adj_ = < 0.0001), and *leukocyte migration* (*P*_adj_ = < 0.0001) as shown in Figure 2B,C.

From the volcano plot, we next selected proteins 10 proteins for further investigation (CD160, ANGPT1, EPCAM, CCL25, GAL, HSPA1A, IL17RB, TREM2, TNFRSF11B, CXCL1). This included proteins that survived FDR correction and five extra proteins (based on their *p*‐value), allowing some flexibility, to address the reduced statistical power. To further understand the potential roles of these proteins in disease processes we conducted the same enrichment analysis. Findings aligned with previous description, with the protein of interest involved in the same processes (Figure 2D).

To better understand the cell‐type specificity of the 10 candidate proteins, we analyzed their percentage of mRNA expression across different cell types using single‐cell transcriptomics data from the Allen Human Brain Atlas (Figure 2E). The analysis revealed that most proteins showed moderate to strong correlations with non‐neuronal populations specifically astrocytes, as well as neuronal populations, including both GABAergic and glutamatergic neurons. Some proteins displayed distinct cell‐type associations: CXCL1 and HSPA1A exhibited particularly strong correlations with endothelial cells, while HSPA1A also showed a notable correlation with microglia, alongside TREM2. Proteins like TNFRSF11B and IL17RB had stronger associations with OPCs. No significant correlations were observed with oligodendrocytes.

### 3.4 Protein distribution increased by TSPO PET binding

We then build a Spearman correlation analysis, visualized it in a sunburst chart, to explore the relationship between inflammatory and AD imaging biomarkers (Figure 2F). This revealed high inter‐protein correlation [ANGPT1 and IL17RB (*r* = 0.79, *p* < 0.001), CXCL1 and ANGPT1 (*r* = 0.74, *p* < 0.001), and HSPA1A and TNFRSF11B (*r* = 0.83, *p* < 0.001)], moderate to strong correlations with TSPO PET [TNFRSF11B (*r* = 0.57, *p* < 0.001), CXCL1 (*r* = 0.52, *p* < 0.001), and HSPA1A (*r* = 0.40, *p* < 0.001)], and lower correlation with amyloid and tau PET [amyloid PET and ANGPT1 (*r* = 0.27, *p* = 0.008), amyloid PET and HSPA1A (*r* = 0.39, *p* < 0.001), tau PET and ANGPT1 (*r* = 0.33, *p* < 0.001), tau PET and TNFRSF11B (*r* = 0.27, *p* = 0.002)]. Furthermore, participants were segregated into TSPO PET quartile groups, and the CSF protein distribution was visualized with boxplots. The expression of these proteins increased in a stepwise manner from lower to higher TSPO PET signal, with all TSPO groups having higher protein levels as compared with the first quartile group. Besides, comparative correlation analysis revealed that CXCL1 and TNFRSF11B exhibited significantly stronger correlations with TSPO PET compared to Amyloid PET and Tau PET (CXCL1: *p* < 0.05, TNFRSF11B: *p* < 0.01) (Table S4), as well as independently from amyloid and tau PET (Table S5). Based on more detailed analysis (both unadjusted and adjusted for covariates), including survival through quartile‐based assessment and evaluation of TSPO PET as a continuous variable, as well as their strong correlation with TSPO PET, we identified and retained two final proteins of interest, namely, TNFRSF11B and CXCL1. More detailed results of the linear models can be found in the Table S6.

### 3.5 Chord diagram showed associations between CSF proteins and TSPO PET uptake

**FIGURE 3:**
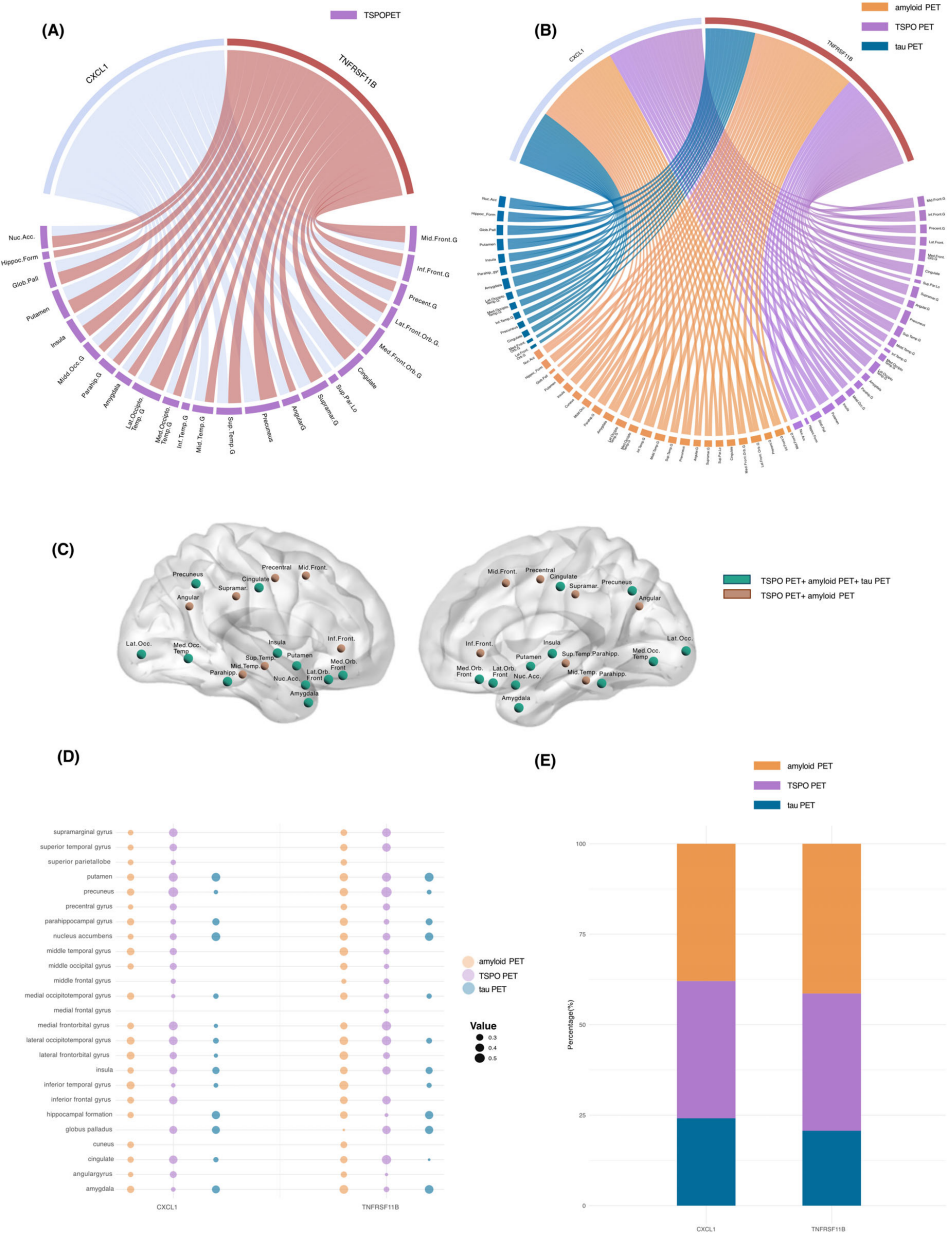
(A) Chord diagram showing correlation of two selected CSF proteins CXCL1, and TNFRSF11 and *TSPO PET* uptake in 45 anatomical brain regions (only significant correlations are displayed). (B) Chord diagram showing correlation of CSF proteins CXCL1 and TNFRSF11 and *TSPO*, amyloid and tau PET uptake in 45 anatomical brain regions (only significant correlations are displayed). (C) Visual colocalization of proteins in the brain regions (*TSPO PET* + amyloid PET and tau PET in green and *TSPO PET* + amyloid PET in orange). (D) Scatter plot illustrating the correlation values for each brain region and protein, categorized by PET tracer. (E) Bar plot displaying the proportions (percentage) of PET types (amyloid PET, TSPO PET and tau PET) for the proteins of interest (CXCL1 and TNFRSF11B). CFS, cerebrospinal fluid; PET, positron emission tomography; TSPO, translocator protein.

To further understand the relationship of these proteins with the imaging outcomes, correlated the protein levels of TNFRSF11B and CXCL1 with TSPO PET uptake in 45 anatomically defined brain regions (Figure 3A). Out of these, 23 brain regions had TSPO PET uptake significantly correlated with CXCL1 and TNFRSF11B, which interestingly includes regions highly involved in AD, such as the hippocampus, amygdala, and putamen. The number of significant correlations between the CSF proteins and TSPO PET binding, in the different brain regions, were similar across the proteins evaluated. The results of the FDR‐corrected analyses were consistent with the uncorrected findings (Figure S2A).

#### 3.5.1 Chord diagram showing colocalized correlations with TSPO, amyloid or tau PET

The previous correlation analysis, between PET uptake and CSF inflammatory proteins (CXCL1 and TNFRSF11B), was then repeated but now including amyloid and tau PET load, in the same 45 brain anatomical regions (Figure 3B,C). Similarly, as before, were identified significant associations with PET in 23 brain regions, including areas implicated in AD pathology, such as hippocampus, amygdala, putamen, nucleus accumbens, parahippocampal gyrus, insula, and cingulate. Once again, the correlations between a given CSF protein and the uptake of the different PET tracers were similar and consistent across all two proteins. The results of the FDR‐corrected analyses were consistent with the uncorrected findings (Figure 2B,C).

However, unique correlations were also identified. CXCL1 was the only protein correlating to TSPO PET uptake in the superior parietal lobe and inferior temporal gyrus, while TNFRSF11B was the only protein to correlate with amyloid PET load in the globus pallidus (Figure 3D).

Despite the similar pattern of correlations across proteins, for a single protein, the correlations were more frequently observed with amyloid and TSPO PET (23 brain regions), and a smaller proportion with tau PET (14 brain regions). Of all significant correlations, a single protein had, on average, 37.9% of them were with TSPO PET uptake in the different brain region, 39.6% with amyloid PET, and 22.5% with tau PET (Figure 3E). Notably, the brain regions significantly associated with inflammatory proteins were often shared among TSPO and amyloid PET, and in some cases among TSPO, amyloid, and tau, indicating overlap in the regions impacted by these pathological processes (Figure 3C).

### 3.6 Plasma proteins not found to be differentially expressed according to TSPO PET status

We repeated the CSF analyses now with plasma data. First, we determined the DEPs according to the TSPO PET status. LIMMA analyses, adjusted for age and brain amyloid load (PET SUVR), unveiled no proteins that remained statistically significant (*p* < 0.05) after FDR correction. Detailed results of the plasma marker analysis are provided in Figure S3A and Table S7.

Next, we tested whether the candidate CSF proteins, found in the initial analyses, were correlated with the proteins quantified in plasma. This analysis unveiled distinct patterns of positive and negative correlations (Figure S3B). Interestingly, MVK and ENPP7 were positively correlated with most of the CSF protein candidates whereas TNFRSF13B and CCL28 corelated negatively with most of them.

### 3.7 Analytical performance of immune‐related proteins quantification

To investigate the possible differences between CSF and plasma findings, we evaluated the performance of the Olink assay in both plasma and CSF matrices by showing the assays with less than 70% detectability. This visualization highlighted the variability in assay performance. Proteins such as BACH1, BID, CASP2, IL10, and IL4 showed no detectability (0%) in CSF (Figure S4A), while DGKZ, IL2, ALDH3A1, IL24, and IL13 showed no detectability (0%) in plasma (Figure S4B). Moreover, we observed that proteins with NPX levels below the limit of detection (LOD) were more prevalent in CSF than in plasma samples (109 proteins with NPX values below LOD in plasma, 324 proteins with NPX values below LOD in CSF). In CSF, 156 targets had detectability above 75%, 189 above 50%; in plasma, 56 targets had detectability above 75%, and 69 above 50%. Additionally, we identified common assays with > 50% NPX values below LOD in both plasma and CSF matrices, indicating potential issues with these specific protein quantifications across sample types that may require further investigation or optimization (Figure S4C). It is noteworthy to mention that the proteins identified in our findings had high detectability (100% for CD160, CXCL1, EPCAM, TNFRSF11B, and TREM2; 99% for GAL and HSPA1A; 94% for ANGPT1 and IL17RB; > 71% CCL25).

## 4 DISCUSSION

The immense progress in identifying biofluid biomarkers for AD, detectable in both CSF and blood, provides optimism that similar advancements can be made for other unmet needs in the molecular phenotyping of neurodegenerative disorders. One such need is for inflammation biomarkers, given this biological process is widely implicated in AD and has long served as a therapeutic target.^46^ This exploratory work focused on inflammation‐related proteins in biofluids, particularly in the CSF, and evaluated their associations with TSPO PET, used as a proxy for neuroinflammation.^32^ We demonstrated significant associations between TSPO PET binding and certain inflammatory proteins in the CSF, suggesting a direct link between neuroinflammation and the expression of these proteins, potentially implicating them in the pathogenic processes underlying AD. Importantly, we could not replicate these findings in blood, suggesting that inflammation is highly sensitive to body compartmentalization.

In AD, neuroinflammation is characterized by the glial responses in the CNS and has been defined as a contributor in the pathophysiology of the disease.^47^ However, its precise role is still under debate. For this reason, it is important to screen and explore biomarkers of neuroinflammation across different disease stages to identify protein patterns that could better explain their pathological role.^48^, ^49^

FEA of DEPs in the CSF of high TSPO PET binders provided insights into which BP are associated with AD and neuroinflammation. Processes such as the positive regulation of MAPK cascade, highly involved with microglia‐mediated neuroinflammation in AD,^50^, ^51^ positive regulation of ERK1 and ERK2 cascade, suggested to be one of the most upregulated phosphoproteins within the MAPK pathway,^52^, ^53^ cytokine‐mediated signaling pathway,^54^, ^55^ and leukocyte migration^56^ have been repeatedly implicated in AD pathology, highlighting the interconnection between inflammation and neuronal dysfunction in the disease.

The inclusion of proteins that did not pass FDR correction reflects the exploratory nature of this study, aimed at generating potential leads for future research. This approach should be interpreted with caution and is not intended to identify definitive biomarkers of inflammation but rather to highlight candidates for further validation. Indeed, these proteins have been previously linked to pathological processes present in AD, for example, microglial modulation and neuroprotection, which confirms their potential importance in future research scenario. TNF Receptor Superfamily Member 11B (TNFRSF11B), interacts with inflammatory mediators (TNF‐α and cytokines). A microarray‐based transcriptomic study in the hippocampus of AD patients reported its upregulation.^57^ Furthermore, an interaction between the TNFRSF1B variant rs976881 and CSF sTNFR2 levels has been found to influence cognitive decline in AD.^58^ C‐X‐C Motif Chemokine Ligand 1 (CXCL1) was reported to have significantly higher levels in monocytes from AD patients.^59^ Additionally, other studies have shown that this protein can induce caspase‐3‐dependent tau cleavage, leading to the abnormal extracellular distribution of truncated tau protein.^60^

The cell‐type‐specific expression of these proteins unveiled valuable insights. The association of TNFRSF11B and ANGPT1 with astrocytes, and of HSAPA1A with the microglia, suggest that these proteins may be crucial in astrocytic and microglial activation, crucial for Aβ protein clearance and inflammatory responses.^61^ The strong correlation of CXCL1 and HSPA1A with endothelial cells showed their importance in maintaining the blood–brain barrier (BBB) integrity,^62^ a crucial process in AD. Besides, TNFRSF11B's association with OPCs suggests a role in the Aβ plaque environment, where OPCs may develop an inflammatory state and may be no longer able to mature into fully functional oligodendrocytes.^63^

To understand the complex network of interactions among these inflammatory biomarkers, we performed a Spearman cross‐correlation matrix, showing a high degree of inter‐protein correlation, indicating a potential collective contribution to the BP underlying AD. Therefore, these proteins could be part of a coordinated response of the immune system in AD and may be regulated by common upstream factors or pathways or part of a series of signaling cascades within cells, responding to pathological changes in the brain. Furthermore, the stepwise increase in the protein expression from lower to higher TSPO PET binding suggests that these proteins increase as the neuroinflammation intensifies and that these targets in the CSF could be involved in the neuroinflammatory process in the context of AD. Of note, the comparative correlation results suggested their potential relevance in differentiating TSPO PET‐related inflammation independently of amyloid and tau pathologies

Significant correlations between selected CSF inflammatory proteins and TSPO PET binding across brain regions involved in AD were also reported (hippocampus, amygdala, putamen, nucleus accumbens, parahippocampal gyrus, insula, and cingulate). These brain regions are acknowledged for their involvement in cognitive and emotional processes and linked to AD‐related changes. Importantly, these areas show a notable enrichment of microglia and astrocytes, cells responsible of neuroinflammatory responses, and oligodendrocytes, crucial for preserving myelin integrity.^64^ This spatial consistency supports the hypothesis that these neuroinflammatory proteins and the underlying AD processes converge in these regions and could therefore play a role in AD progression. Moreover, the consistent associations observed in regions implicated in AD pathology across different PET modalities (amyloid, tau, and TSPO) may indicate a chronological progression of AD. The higher proportions of associations with amyloid and TSPO PET compared to tau PET might indicate that neuroinflammation may be an earlier event in AD pathology, potentially preceding overt tau pathology typically emerging at later stages. Of note, the distribution of the participants of the cohort (13 participants tau PET‐positive who were also amyloid PET‐positive), among the 35 amyloid‐positive participants, suggests that most participants were in an earlier, amyloid‐predominant stage of AD. The smaller proportion of correlations observed with tau PET could reflect the dynamic progression of pathology, where tau accumulation typically becomes more prominent and spatially widespread in later stages of the disease. Correlations with tau PET in our study may therefore represent early downstream effects of amyloid deposition or initial tau‐related neuroinflammatory processes.

Identifying inflammation‐related proteins in plasma, which requires differentiating central from peripheral immune response, is not an easy task but this challenge needs addressing given the extreme importance of using blood biomarkers in the current AD research scenario. Of note, blood assays are highly preferable than CSF, as they are less invasive, less costly and more accessible for clinical use. Importantly, GFAP has been recently added to the NIA‐AA Revised Clinical Criteria as a biomarker of neuroinflammation in AD, paving the way for the future progress in the scenario of blood‐based biomarkers of inflammation. Besides, validated blood biomarkers of immune‐related proteins could be a tool to monitor the inflammatory response to anti‐amyloid treatments. This scenario of complexity could be grasped with the correlations of CSF and plasma proteins, unveiling patterns of positive and negative correlations and indicating indicate different regulatory mechanisms between the central and peripheral compartments. The positive associations with MVK, an enzyme essential for the mevalonate pathway and linked to metabolic disorders, could support the important role of metabolic pathways, and neuroinflammatory processes in the disease process.^65^, ^66^ Furthermore, the positive correlation with ENPP7, a substrate for beta‐secretase (BACE1) involved in amyloid‐beta plaque formation,^67^ may imply that increased activity of this substrate, together with other proteins could be connected to increased amyloid deposition. In contrast, the negative correlations with TNFRSF13B and CCL28 could indicate a reduced activity in pathways related to immune modulation and chemotaxis possibly as a counter‐regulatory response. Together, these patterns underscore the multifaceted nature of AD pathology. Importantly, while plasma offered a broader range of detectable proteins, fewer proteins passed the 75% detectability threshold, potentially impacting data quality and statistical power. The lower detectability of proteins in plasma, compared to CSF, may have limited the ability to detect meaningful associations, highlighting the need for more sensitive methods.

This study is not without limitations. Despite the largest study of its kind, with > 100 participants with amyloid‐, tau‐, TSPO PET and CSF biomarkers, these findings warrant more statistical power to be definitive. Moreover, there are some intrinsic limitations of the methods to consider. TSPO PET reflects microglial and astrocyte reactivity but is also expressed in endothelial cells and its properties as a radiopharmaceutical are modest (low binding affinity, high nonselective binding, and high lipophilicity).^68^ However, the patients included in our cohort consisted in selected high affinity binders, which is a strength of the study. Furthermore, there is the need for standardization across studies and tracers, that can affect the comparability of the results. Regarding the proteomic data, the Olink technology offers high‐throughput protein quantification, but it is not exempt of limitations. As previously discussed, several proteins had NPX value below the LOD in both biological matrices. Additionally, the panel of proteins assessed by Olink may not cover all relevant biomarkers of interest. Moreover, protein quantification was assessed at a single timepoint, but inflammation is a dynamic process and evolves over time,^69^ therefore the need of longitudinal studies. Inflammation is a complex scenario, and the association between AD and age underscored the complexity in dissociating disease‐specific factors from those related to aging. This represented a challenge we faced when attempting to distinguish amyloid‐related processes from age‐related inflammation. Furthermore, it is challenging to obtain other independent cohorts with comparable clinical, imaging, and proteomic data, to validate these findings.

In summary, our study provides evidence linking neuroinflammation, as measured by TSPO PET, with specific CSF inflammatory proteins in the AD scenario. The high inter‐protein correlations, combined with significant associations across brain regions, underscore the complex network of interactions driving AD pathology. These findings highlight the importance of targeting neuroinflammation in therapeutic strategies and suggest that the selected proteins could be potentially valuable biomarkers for monitoring disease progression and treatment efficacy. Notably, we did not find any associations of TSPO PET data with blood neuroinflammatory biomarkers, with different patterns of positive and negative associations between CSF and plasma proteins, highlighting the complexity of immune responses in the brain‐body interactions.

## CONFLICT OF INTEREST STATEMENT

Nicholas J. Ashton has served as consultant for Quanterix and has given lectures in symposia sponsored by Lilly, Quanterix and Biogen. Joseph Therriault has received consulting fees from the Neurotorium educational platform and from Alzheon Inc., outside of the scope of this work. Kaj Blennow has served as a consultant, at advisory boards, or at data monitoring committees for Acumen, Abcam, ALZ‐ path, AriBio, Axon, BioArctic, Biogen, Eisai, JOMDD/Shimadzu, Julius Clinical, Lilly, MagQu, Novartis, Ono Pharma, Pharmatrophix, Prothena, Roche Diagnostics, and Siemens Healthineers, and is a co‐ founder of Brain Biomarker Solutions in Gothenburg AB (BBS), which is a part of the GU Ventures Incubator Program, outside the work presented in this paper. Henrik Zetterberg has served at scientific advisory boards and/or as a consultant for Abbvie, Acumen, Alector, Alzinova, ALZPath, Annexon, Apellis, Artery Therapeutics, AZTherapies, Cognito Therapeuthics, CogRx, Denali, Eisai, Nervgen, Novo Nordisk, Optoceutics, Passage Bio, Pinteon Therapeutics, Prothena, Red Abbey Labs, reMYND, Roche, Samumed, Siemens Healthineers, Triplet Therapeutics, and Wave, has given lectures in symposia sponsored by Cellectricon, Fujirebio, Alzecure, Biogen, and Roche, and is a co‐ founder of Brain Biomarker Solutions in Gothenburg AB (BBS), which is a part of the GU Ventures Incubator Program (outside sub‐ mitted work). All other authors report no disclosures. Eduardo R. Zimmer has served in the scientific advisory board of Nintx, Novo Nordisk and masima. He is also a co‐founder and a minority shareholder at masima. The other authors report no conflicts of interest. Author disclosures are available in the supporting information.

## CONSENT STATEMENT

All participants provided written informed consent, and ethical approval was obtained by the Douglas Mental Health University Institute Research Ethics Board (MP‐18‐2017‐157) and the Montreal Neurological Institute PET working committee.

## Supporting information



Supporting Information

Supporting Information

Supporting Information
