# Supplementary material for: Exploring inflammation‐related protein expression and its relationship with TSPO PET in Alzheimer's disease
Source: Alzheimers Dement. 2025 Apr 28;21(4):e70171. doi: 10.1002/alz.70171 (PMC12035552; doi:10.1002/alz.70171)
Supplement: Supplementary file 1 — Supporting Information [file ALZ-21-e70171-s001.docx]

**Supplemental figure 1:** Composite mask generated from the brain regions that remained significant (in purple) after correction for multiple comparisons using random field theory (REF), utilized for ROI SUVR extraction.

**Supplemental Figure 2. A)** Chord diagram showing correlation of CSF proteins CXCL1, and TNFRSF11 and TSPO PET uptake in 45 anatomical brain regions (only significant correlations after FDR correction are displayed). **B)** Chord diagram showing correlation of CSF proteins CXCL1 and TNFRSF11 and TSPO, amyloid and tau PET uptake in 45 anatomical brain regions (only significant correlations after FDR correction are displayed). **C)** Overlap of brain regions significant in FDR-corrected and uncorrected analysis, shown in a Venn diagram. Each diagram represents a PET imaging modality.

**Supplemental Figure 3. A)** Volcano plot showing the association among 368 plasma proteins in TSPO PET positive versus negative participants by displaying the log2 fold change of protein abundance in plasma (x-axis) against statistical significance (y-axis) between individuals positive and negative for TSPO PET. **B)** Heatmap showing the correlation of the 10 proteins of interest in the CSF and all the plasma proteins. Only significant correlations are displayed, with red gradient color indicating positive associations and blue gradient color indicating negative associations.

**Supplemental Figure 4. A)** Bar plots showing the target proteins having < 70 % detectability (NPX values below the LOD) in CSF samples. The target proteins (y axis) are ordered from lower detectability (0%) to higher detectability (70%). The gradient color scale represents NPX values below the LOD for darker colors. **B)** Bar plots showing the target proteins having < 70 % detectability (NPX values below the LOD). The target proteins (y axis) are ordered from lower detectability (0%) to higher detectability (70%). The gradient color scale represents NPX values below the LOD for darker colors. **C)** Common target proteins with >50% NPX Below LOD in both plasma and CSF.
