# Supplementary material for: Exploring inflammation‐related protein expression and its relationship with TSPO PET in Alzheimer's disease
Source: Alzheimers Dement. 2025 Apr 28;21(4):e70171. doi: 10.1002/alz.70171 (PMC12035552; doi:10.1002/alz.70171)
Supplement: Supplementary file 2 — Supporting Information [file ALZ-21-e70171-s003.docx]

**Supplemental Table 1.** Demographics of the TRIAD participants with plasma measurements included in this study

| **Characteristics** | **Young (N=21)** | **CU- (N=66)** | | **CU+ (N=18)** | | **MCI- (N=12)** | | **MCI+ (N=21)** | | **AD+ (N=18)** | | **Non-AD (N=9)** | |
| --- | --- | --- | --- | --- | --- | --- | --- | --- | --- | --- | --- | --- | --- |
| **Age, years** | 23 (± 1.6) | 69 (± 9.6) | | 73 (± 5.2) | | 73 (± 5.5) | | 71 (± 8.6) | | 68 (± 9.2) | | 67 (± 7.5) | |
| **Female, n (%)** | 12 (57.1%) | 52 (78.8%) | | 16 (88.9%) | | 5 (41.7%) | | 10 (47.6%) | | 9 (50.0%) | | 5 (55.6%) | |
| **Aβ-PET (SUVR)** | 1.2 (± 0.07) | 1.3 (± 0.10) | | 2.1 (± 0.42) | | 1.3 (± 0.17) | | 2.4 (± 0.36) | | 2.5 (± 0.40) | | 1.2 (± 0.12) | |
| **Tau-PET (SUVR)** | 0.82 (± 0.08) | 0.83 (± 0.08) | | 0.92 (± 0.09) | | 0.82 (± 0.10) | | 1.3 (± 0.39) | | 2.2 (± 0.97) | | 0.80 (± 0.09) | |
| **TSPO-PET (SUVR)** | 0.98 (± 0.04) | 1.1 (± 0.08) | | 1.1 (± 0.12) | | 1.1 (± 0.12) | | 1.2 (± 0.10) | | 1.3 (± 0.12) | | 1.2 (± 0.11) | |
| **TSPO-positive, n (%)** | 0 (0%) | 32 (48.5%) | | 10 (55.6%) | | 6 (50.0%) | | 16 (76.2%) | | 18 (100%) | | 5 (55.6%) | |
|  |  |  |  | |  | |  | |  | |  | |  |

“+“: amyloid-β positive; “-“: amyloid-β negative; Aβ: amyloid-β; AD: Alzheimer’s disease; CU: cognitively unimpaired; MCI: mild cognitively impaired; Non-AD: Non-Alzheimer’s disease; PET: positron emission tomography; SUVR: standard uptake value ratio; TSPO: Translocator protein

**Supplemental Table 2.** Demographics based on other characteristics, including amyloid status and cognitive status, of the TSPO PET positive and negative groups

|  | **TSPO PET + (N=49)** | **TSPO PET - (N=48)** |
| --- | --- | --- |
| **Age, mean (SD)** | 70 (± 6.9) | 51 (± 23) |
| **Sex, n (% TSPO+)** |  |  |
| **female** | 25 (42.4%) | 34 (57.6%) |
| **male** | 24 (63.2%) | 14 (36.8%) |
| **MMSE, mean (SD)** | 27 (3.9) | 29.4 (1) |
| **Disease Group, n (% TSPO +/-)** |  |  |
| **Young** | 0 (0.0 %) | 19 (100.0 %) |
| **CU-** | 15 (53.6 %) | 13 (46.4 %) |
| **CU+** | 4 (40.0 %) | 6 (60.0 %) |
| **MCI-** | 4 (57.1 %) | 3 (42.9 %) |
| **MCI+** | 11 (73.3 %) | 4 (26.7 %) |
| **AD+** | 10 (100.0 %) | 0 (0 %) |
| **Non-AD** | 5 (62.5%) | 3 (37.5 %) |
| **Amyloid status, n (% TSPO +/-)** |  |  |
| **Positive** | 25 (71.4%) | 10 (28.6%) |
| **Negative** | 24 (38.7%) | 38 (61.3%) |
|  |  |  |

“+“: amyloid-β positive; “-“: amyloid-β negative; Aβ: amyloid-β; AD: Alzheimer’s disease; CU: cognitively unimpaired; MCI: mild cognitively impaired; MMSE: Mini-Mental State Examination; Non-AD: Non-Alzheimer’s disease; PET: positron emission tomography; SUVR: standard uptake value ratio; TSPO: Translocator protein

**Supplemental Table 3:** Differentially expressed proteins CSF. The table represents the results of the LIMMA model. From the top, to the bottom, top-ranked genes with adjusted p-values (adj.P.Val), log-fold changes (logFC), and associated gene identifiers (UniProt, Protein) are displayed. Significant genes were identified using false discovery rate (FDR) adjustment (adjust = "fdr") and prioritized by statistical strength (B statistic). First 50 results are displayed.

| **Protein** | **adj.P.Val** | **P.value** | **t** | **B** | **logFC** | **UniProt** |
| --- | --- | --- | --- | --- | --- | --- |
| **CD160** | 0.0424108 | 0.0001309 | 3.9595964 | 0.9099581 | 0.7526814 | O95971 |
| **ANGPT1** | 0.0424108 | 0.0002581 | 3.7711082 | 0.3356697 | 0.6933856 | Q15389 |
| **EPCAM** | 0.0424108 | 0.0003457 | 3.687498 | 0.0785485 | 0.7435386 | P16422 |
| **CCL25** | 0.0467783 | 0.0005761 | 3.5401808 | -0.3599125 | 0.6275038 | O15444 |
| **GAL** | 0.0467783 | 0.0006356 | 3.5122105 | -0.4434608 | 0.6977294 | P22466 |
| **IL17RB** | 0.0539759 | 0.00088 | 3.4131477 | -0.7230332 | 0.5924728 | Q9NRM6 |
| **TREM2** | 0.057866 | 0.0012369 | 3.3100572 | -1.0136253 | 0.5474923 | Q9NZC2 |
| **HSPA1A** | 0.057866 | 0.0012837 | 3.2993656 | -1.0499311 | 0.57862 | P0DMV8 |
| **TNFRSF11B** | 0.057866 | 0.0014745 | 3.2559626 | -1.1648388 | 0.564148 | O00300 |
| **CXCL1** | 0.057866 | 0.0015853 | 3.2354641 | -1.2230024 | 0.587122 | P09341 |
| **SIGLEC1** | 0.057866 | 0.0017886 | 3.1976982 | -1.3242343 | 0.6061107 | Q9BZZ2 |
| **VEGFD** | 0.057866 | 0.0021053 | 3.1449397 | -1.4637985 | 0.5349645 | O43915 |
| **FST** | 0.057866 | 0.002242 | 3.1261281 | -1.5146369 | 0.5112093 | P19883 |
| **IL1B** | 0.057866 | 0.0022245 | 3.1313117 | -1.5205594 | 0.6801614 | P01584 |
| **B4GALT1** | 0.057866 | 0.0023587 | 3.1092955 | -1.5605496 | 0.5859467 | P15291 |
| **EPHA1** | 0.0579047 | 0.0025176 | 3.0889526 | -1.6122946 | 0.5317407 | P21709 |
| **PGF** | 0.0594841 | 0.0027479 | 3.0584142 | -1.6939548 | 0.5024094 | P49763 |
| **MZB1** | 0.059977 | 0.0029337 | 3.0405883 | -1.7416238 | 0.6110107 | Q8WU39 |
| **LHPP** | 0.0782584 | 0.0040405 | 2.9318445 | -2.0166373 | 0.4981169 | Q9H008 |
| **SCGB1A1** | 0.0827174 | 0.0046822 | 2.882418 | -2.143606 | 0.4804598 | P11684 |
| **SCRN1** | 0.0827174 | 0.0048817 | 2.8702077 | -2.1656507 | 0.5276743 | Q12765 |
| **ISM1** | 0.0827174 | 0.0049656 | 2.8654191 | -2.1780604 | 0.5197071 | B1AKI9 |
| **ATP5IF1** | 0.0827174 | 0.0051698 | 2.8511988 | -2.2178323 | 0.4963969 | Q9UII2 |
| **CRKL** | 0.0856004 | 0.0059431 | 2.8024702 | -2.334885 | 0.4817353 | P46109 |
| **PRELP** | 0.0856004 | 0.0059836 | 2.7992453 | -2.3459442 | 0.4791512 | P51888 |
| **TIMP3** | 0.0856004 | 0.0061912 | 2.7892538 | -2.3672066 | 0.5368417 | P35625 |
| **FSTL3** | 0.0856004 | 0.0063412 | 2.7791882 | -2.394421 | 0.4724838 | O95633 |
| **CCN2** | 0.0856004 | 0.0065131 | 2.7694919 | -2.4184449 | 0.4726887 | P29279 |
| **IL7** | 0.0876601 | 0.006908 | 2.7515784 | -2.4643907 | 0.5520546 | P13232 |
| **CCL23** | 0.0961562 | 0.0083149 | 2.685762 | -2.6219628 | 0.4776384 | P55773 |
| **HCLS1** | 0.0961562 | 0.0082763 | 2.6865982 | -2.6241044 | 0.5298268 | P14317 |
| **IL18** | 0.0961562 | 0.0083614 | 2.6821726 | -2.6261505 | 0.5072156 | Q14116 |
| **FGF19** | 0.0996951 | 0.0093061 | 2.6458932 | -2.6968169 | 0.5042271 | O95750 |
| **DAG1** | 0.0996951 | 0.0093257 | 2.6431947 | -2.711112 | 0.4630596 | Q14118 |
| **MEPE** | 0.0996951 | 0.009752 | 2.6290844 | -2.7475002 | 0.6018176 | Q9NQ76 |
| **CCL4** | 0.0996951 | 0.0097528 | 2.6286583 | -2.7480435 | 0.5879037 | P13236 |
| **PDLIM7** | 0.1013004 | 0.0104865 | 2.601561 | -2.8236594 | 0.4578125 | Q9NR12 |
| **ANGPTL4** | 0.1013004 | 0.0107356 | 2.5922994 | -2.8271825 | 0.4928098 | Q9BY76 |
| **CCL7** | 0.1013004 | 0.0107004 | 2.596189 | -2.8279562 | 0.5685322 | P80098 |
| **ERBB3** | 0.1073836 | 0.0116721 | 2.5617292 | -2.9014419 | 0.4742263 | P21860 |
| **IL1R2** | 0.1105131 | 0.0123126 | 2.5427488 | -2.9379766 | 0.4473444 | P27930 |
| **DBNL** | 0.118233 | 0.0137137 | 2.5027201 | -3.0255379 | 0.4587452 | Q9UJU6 |
| **TLR3** | 0.118233 | 0.0138153 | 2.4989965 | -3.0365859 | 0.4969174 | O15455 |
| **MMP10** | 0.1197977 | 0.0143236 | 2.4861073 | -3.0668936 | 0.4792076 | P09238 |
| **CDON** | 0.1237784 | 0.0153792 | 2.4593352 | -3.1186288 | 0.445742 | Q4KMG0 |
| **LIFR** | 0.1237784 | 0.0158087 | 2.4489084 | -3.1421013 | 0.4878416 | P42702 |
| **SCGB3A2** | 0.1237784 | 0.0155716 | 2.4549444 | -3.1475074 | 0.4444793 | Q96PL1 |
| **IL17D** | 0.1273043 | 0.0166049 | 2.4311574 | -3.1772207 | 0.4878543 | Q8TAD2 |
| **CXCL9** | 0.1343343 | 0.0178869 | 2.4017633 | -3.2433308 | 0.4113441 | Q07325 |
| **OMD** | 0.1352729 | 0.0187471 | 2.3834059 | -3.2879136 | 0.4869849 | Q99983 |

**Supplemental Table 4:** The table presents Pearson correlation coefficients comparing the strength of association between each protein and the global PET SUVR measures for each of the tracers. The correlations for each protein with the specified PET imaging types are displayed alongside their p-values (P.value). The Significance column indicates the level of statistical significance, with * denoting p < 0.05 and ** denoting p < 0.01. Proteins with significant differences in correlations are highlighted, indicating a stronger association with TSPO PET compared to Amyloid PET or Tau PET.

| **Protein** | **PET type 1** | **PET type 2** | **Correlation** | **P.value** | **Significance** |
| --- | --- | --- | --- | --- | --- |
| **ANGPT1** | TSPO PET | Amyloid PET | 0.2334 | 0.1411 | NS |
| **ANGPT1** | TSPO PET | Tau PET | 0.2337 | 0.1416 | NS |
| **CCL25** | TSPO PET | Amyloid PET | 0.1424 | 0.0579 | NS |
| **CCL25** | TSPO PET | Tau PET | 0.1028 | 0.0318 | * |
| **CD160** | TSPO PET | Amyloid PET | 0.2226 | 0.2698 | NS |
| **CD160** | TSPO PET | Tau PET | 0.2064 | 0.2263 | NS |
| **CXCL1** | TSPO PET | Amyloid PET | 0.2109 | 0.0261 | * |
| **CXCL1** | TSPO PET | Tau PET | 0.1820 | 0.0159 | * |
| **EPCAM** | TSPO PET | Amyloid PET | 0.0397 | 0.0643 | NS |
| **EPCAM** | TSPO PET | Tau PET | 0.1711 | 0.3080 | NS |
| **GAL** | TSPO PET | Amyloid PET | 0.2272 | 0.1747 | NS |
| **GAL** | TSPO PET | Tau PET | 0.2175 | 0.1553 | NS |
| **HSPA1A** | TSPO PET | Amyloid PET | 0.3316 | 0.2878 | NS |
| **HSPA1A** | TSPO PET | Tau PET | 0.2802 | 0.1564 | NS |
| **IL17RB** | TSPO PET | Amyloid PET | 0.1490 | 0.2641 | NS |
| **IL17RB** | TSPO PET | Tau PET | 0.1921 | 0.4010 | NS |
| **TNFRSF11B** | TSPO PET | Amyloid PET | 0.2721 | 0.0215 | * |
| **TNFRSF11B** | TSPO PET | Tau PET | 0.1848 | 0.0040 | ** |
| **TREM2** | TSPO PET | Amyloid PET | 0.1556 | 0.0708 | NS |
| **TREM2** | TSPO PET | Tau PET | 0.2056 | 0.1379 | NS |

**Supplemental Table 5:** The table presents partial correlation analysis assessing the relationship between the proteins and TSPO PET while controlling for the potential confounding effects of amyloid PET and tau PET. The partial correlations for each protein are displayed alongside their p-values (P.value).

| **Protein** | **Partial Correlation** | **P.value** |
| --- | --- | --- |
| **TNFRSF11B** | 0.4704 | 2.00E-06 |
| **CXCL1** | 0.4549 | 6.70E-06 |
| **CCL25** | 0.4266 | 2.50E-05 |
| **ANGPT1** | 0.4259 | 2.90E-05 |
| **HSPA1A** | 0.4238 | 2.60E-05 |
| **CD160** | 0.3678 | 3.90E-04 |
| **GAL** | 0.366 | 3.90E-04 |
| **TREM2** | 0.3358 | 1.00E-03 |
| **EPCAM** | 0.2799 | 7.20E-03 |
| **IL17RB** | 0.2777 | 7.00E-03 |

**Supplemental Table 6:** This table summarizes the results of the linear model analysis, including the effect size (Estimate), standard error (Std. Error), t-statistic (T.value), and p-value (P.value) for each variable. The Significance column indicates the statistical significance of the results, with levels denoted as follows: *** (p < 0.001), ** (p < 0.01), and * (p < 0.05). Variables without asterisks are not statistically significant. The tables represent the following linear models:

- MODEL 1: Top 10 proteins, ~ TSPO PET + Age + Sex + Amyloid PET
- MODEL 2: Top 10 proteins, ~ TSPO PET quartiles + Age + Sex + Amyloid PET
- MODEL 3: Top 10 proteins, ~ TSPO PET + Age + Sex
- MODEL 4: Top 10 proteins, ~ TSPO PET quartiles + Age + Sex

MODEL 1

| **Protein** | **Term** | **Estimate** | **Std.Error** | **T.value** | **P.value** | **Significance** |
| --- | --- | --- | --- | --- | --- | --- |
| TREM2 | (Intercept) | -2.1687 | 0.8798 | -2.4650 | 0.0156 | * |
| TREM2 | TSPO PET | 1.5901 | 0.9291 | 1.7114 | 0.0904 |  |
| TREM2 | Age | 0.0162 | 0.0062 | 2.6056 | 0.0107 | * |
| TREM2 | Gender (male) | -0.3280 | 0.2024 | -1.6207 | 0.1085 |  |
| TREM2 | Amyloid PET | -0.2922 | 0.1794 | -1.6286 | 0.1069 |  |
| TNFRSF11B | (Intercept) | -2.9170 | 0.8391 | -3.4762 | 0.0008 | *** |
| TNFRSF11B | TSPO PET | 2.0248 | 0.8862 | 2.2848 | 0.0246 | * |
| TNFRSF11B | Age | 0.0180 | 0.0059 | 3.0427 | 0.0031 | ** |
| TNFRSF11B | Gender (male) | -0.2119 | 0.1930 | -1.0979 | 0.2752 |  |
| TNFRSF11B | Amyloid PET | -0.2311 | 0.1711 | -1.3508 | 0.1801 |  |
| IL17RB | (Intercept) | -2.4437 | 0.8855 | -2.7598 | 0.0070 | ** |
| IL17RB | TSPO PET | 1.3924 | 0.9351 | 1.4890 | 0.1399 |  |
| IL17RB | Age | 0.0158 | 0.0063 | 2.5191 | 0.0135 | * |
| IL17RB | Gender (male) | -0.2694 | 0.2037 | -1.3226 | 0.1893 |  |
| IL17RB | Amyloid PET | 0.0139 | 0.1806 | 0.0768 | 0.9389 |  |
| HSPA1A | (Intercept) | -2.5831 | 0.7888 | -3.2748 | 0.0015 | ** |
| HSPA1A | TSPO PET | 0.8076 | 0.8330 | 0.9695 | 0.3348 |  |
| HSPA1A | Age | 0.0239 | 0.0056 | 4.2852 | 0.0000 | *** |
| HSPA1A | Gender (male) | -0.0166 | 0.1814 | -0.0916 | 0.9272 |  |
| HSPA1A | Amyloid PET | 0.1411 | 0.1608 | 0.8770 | 0.3828 |  |
| GAL | (Intercept) | -2.2831 | 0.9094 | -2.5105 | 0.0138 | * |
| GAL | TSPO PET | 1.5352 | 0.9604 | 1.5985 | 0.1134 |  |
| GAL | Age | 0.0131 | 0.0064 | 2.0388 | 0.0444 | * |
| GAL | Gender (male) | -0.0902 | 0.2092 | -0.4310 | 0.6675 |  |
| GAL | Amyloid PET | -0.1255 | 0.1854 | -0.6770 | 0.5001 |  |
| EPCAM | (Intercept) | -1.9821 | 0.8707 | -2.2764 | 0.0252 | * |
| EPCAM | TSPO PET | 1.1556 | 0.9196 | 1.2567 | 0.2121 |  |
| EPCAM | Age | 0.0197 | 0.0062 | 3.2084 | 0.0018 | ** |
| EPCAM | Gender (male) | -0.1474 | 0.2003 | -0.7359 | 0.4637 |  |
| EPCAM | Amyloid PET | -0.2681 | 0.1776 | -1.5097 | 0.1346 |  |
| CXCL1 | (Intercept) | -2.9638 | 0.8030 | -3.6909 | 0.0004 | *** |
| CXCL1 | TSPO PET | 1.8118 | 0.8481 | 2.1364 | 0.0353 | * |
| CXCL1 | Age | 0.0210 | 0.0057 | 3.6934 | 0.0004 | *** |
| CXCL1 | Gender (male) | -0.3307 | 0.1847 | -1.7902 | 0.0767 |  |
| CXCL1 | Amyloid PET | -0.1359 | 0.1637 | -0.8299 | 0.4088 |  |
| CD160 | (Intercept) | -2.5785 | 0.8746 | -2.9481 | 0.0041 | ** |
| CD160 | TSPO PET | 1.3206 | 0.9237 | 1.4297 | 0.1562 |  |
| CD160 | Age | 0.0169 | 0.0062 | 2.7265 | 0.0077 | ** |
| CD160 | Gender (male) | -0.0078 | 0.2012 | -0.0387 | 0.9692 |  |
| CD160 | Amyloid PET | 0.0346 | 0.1783 | 0.1938 | 0.8468 |  |
| CCL25 | (Intercept) | -1.7401 | 0.7507 | -2.3181 | 0.0227 | * |
| CCL25 | TSPO PET | -0.0951 | 0.7928 | -0.1200 | 0.9047 |  |
| CCL25 | Age | 0.0342 | 0.0053 | 6.4480 | 0.0000 | *** |
| CCL25 | Gender (male) | 0.1420 | 0.1727 | 0.8224 | 0.4130 |  |
| CCL25 | Amyloid PET | -0.1778 | 0.1531 | -1.1613 | 0.2485 |  |
| ANGPT1 | (Intercept) | -2.7338 | 0.8874 | -3.0806 | 0.0027 | ** |
| ANGPT1 | TSPO PET | 1.6469 | 0.9372 | 1.7572 | 0.0822 |  |
| ANGPT1 | Age | 0.0111 | 0.0063 | 1.7767 | 0.0790 |  |
| ANGPT1 | Gender (male) | -0.0678 | 0.2041 | -0.3319 | 0.7407 |  |
| ANGPT1 | Amyloid PET | 0.1398 | 0.1810 | 0.7728 | 0.4417 |  |

MODEL 2

| **Protein** | **Term** | **Estimate** | **Std.Error** | **T.value** | **P.value** | **Significance** |
| --- | --- | --- | --- | --- | --- | --- |
| TREM2 | (Intercept) | -0.4673 | 0.3738 | -1.2501 | 0.2145 |  |
| TREM2 | TSPO quartile 2 | 0.1764 | 0.3698 | 0.4770 | 0.6345 |  |
| TREM2 | TSPO quartile 3 | 0.8662 | 0.3813 | 2.2716 | 0.0255 | * |
| TREM2 | TSPO quartile 4 | 0.8496 | 0.3980 | 2.1349 | 0.0355 | * |
| TREM2 | Age | 0.0104 | 0.0080 | 1.2931 | 0.1993 |  |
| TREM2 | Gender (male) | -0.4067 | 0.1976 | -2.0584 | 0.0425 | * |
| TREM2 | Amyloid PET | -0.2913 | 0.1751 | -1.6643 | 0.0996 |  |
| TNFRSF11B | (Intercept) | -0.8241 | 0.3594 | -2.2927 | 0.0242 | * |
| TNFRSF11B | TSPO quartile 2 | 0.2146 | 0.3556 | 0.6034 | 0.5478 |  |
| TNFRSF11B | TSPO quartile 3 | 0.8515 | 0.3667 | 2.3221 | 0.0225 | * |
| TNFRSF11B | TSPO quartile 4 | 0.9172 | 0.3827 | 2.3967 | 0.0186 | * |
| TNFRSF11B | Age | 0.0133 | 0.0077 | 1.7230 | 0.0884 |  |
| TNFRSF11B | Gender (male) | -0.2714 | 0.1900 | -1.4287 | 0.1566 |  |
| TNFRSF11B | Amyloid PET | -0.2275 | 0.1683 | -1.3517 | 0.1799 |  |
| IL17RB | (Intercept) | -0.9903 | 0.3827 | -2.5879 | 0.0113 | * |
| IL17RB | TSPO quartile 2 | 0.0664 | 0.3786 | 0.1754 | 0.8612 |  |
| IL17RB | TSPO quartile 3 | 0.6545 | 0.3904 | 1.6764 | 0.0972 |  |
| IL17RB | TSPO quartile 4 | 0.6275 | 0.4074 | 1.5402 | 0.1271 |  |
| IL17RB | Age | 0.0125 | 0.0082 | 1.5213 | 0.1317 |  |
| IL17RB | Gender (male) | -0.3221 | 0.2023 | -1.5927 | 0.1148 |  |
| IL17RB | Amyloid PET | 0.0126 | 0.1792 | 0.0701 | 0.9443 |  |
| HSPA1A | (Intercept) | -1.6775 | 0.3414 | -4.9129 | 0.0000 | *** |
| HSPA1A | TSPO quartile 2 | 0.1758 | 0.3378 | 0.5205 | 0.6040 |  |
| HSPA1A | TSPO quartile 3 | 0.6217 | 0.3483 | 1.7848 | 0.0777 |  |
| HSPA1A | TSPO quartile 4 | 0.5432 | 0.3635 | 1.4941 | 0.1387 |  |
| HSPA1A | Age | 0.0186 | 0.0073 | 2.5417 | 0.0128 | * |
| HSPA1A | Gender (male) | -0.0741 | 0.1805 | -0.4106 | 0.6824 |  |
| HSPA1A | Amyloid PET | 0.1465 | 0.1599 | 0.9160 | 0.3621 |  |
| GAL | (Intercept) | -0.6477 | 0.3865 | -1.6759 | 0.0973 |  |
| GAL | TSPO quartile 2 | 0.1262 | 0.3824 | 0.3301 | 0.7421 |  |
| GAL | TSPO quartile 3 | 0.8689 | 0.3943 | 2.2039 | 0.0301 | * |
| GAL | TSPO quartile 4 | 0.7746 | 0.4115 | 1.8825 | 0.0630 |  |
| GAL | Age | 0.0078 | 0.0083 | 0.9380 | 0.3508 |  |
| GAL | Gender (male) | -0.1631 | 0.2043 | -0.7984 | 0.4267 |  |
| GAL | Amyloid PET | -0.1237 | 0.1810 | -0.6835 | 0.4961 |  |
| EPCAM | (Intercept) | -0.7104 | 0.3617 | -1.9641 | 0.0526 |  |
| EPCAM | TSPO quartile 2 | 0.1248 | 0.3579 | 0.3486 | 0.7282 |  |
| EPCAM | TSPO quartile 3 | 0.9866 | 0.3690 | 2.6738 | 0.0089 | ** |
| EPCAM | TSPO quartile 4 | 0.6361 | 0.3851 | 1.6517 | 0.1021 |  |
| EPCAM | Age | 0.0131 | 0.0078 | 1.6913 | 0.0943 |  |
| EPCAM | Gender (male) | -0.2189 | 0.1912 | -1.1451 | 0.2552 |  |
| EPCAM | Amyloid PET | -0.2564 | 0.1694 | -1.5139 | 0.1336 |  |
| CXCL1 | (Intercept) | -1.0415 | 0.3385 | -3.0769 | 0.0028 | ** |
| CXCL1 | TSPO quartile 2 | 0.2830 | 0.3349 | 0.8451 | 0.4003 |  |
| CXCL1 | TSPO quartile 3 | 0.9582 | 0.3453 | 2.7749 | 0.0067 | ** |
| CXCL1 | TSPO quartile 4 | 0.9550 | 0.3604 | 2.6498 | 0.0095 | ** |
| CXCL1 | Age | 0.0142 | 0.0073 | 1.9486 | 0.0545 |  |
| CXCL1 | Gender (male) | -0.4060 | 0.1789 | -2.2695 | 0.0257 | * |
| CXCL1 | Amyloid PET | -0.1285 | 0.1585 | -0.8103 | 0.4199 |  |
| CD160 | (Intercept) | -1.1360 | 0.3702 | -3.0683 | 0.0029 | ** |
| CD160 | TSPO quartile 2 | 0.6600 | 0.3663 | 1.8019 | 0.0749 |  |
| CD160 | TSPO quartile 3 | 1.1963 | 0.3777 | 3.1675 | 0.0021 | ** |
| CD160 | TSPO quartile 4 | 0.8620 | 0.3942 | 2.1869 | 0.0314 | * |
| CD160 | Age | 0.0054 | 0.0080 | 0.6729 | 0.5028 |  |
| CD160 | Gender (male) | -0.0525 | 0.1957 | -0.2685 | 0.7889 |  |
| CD160 | Amyloid PET | 0.0815 | 0.1734 | 0.4699 | 0.6396 |  |
| CCL25 | (Intercept) | -1.7150 | 0.3200 | -5.3595 | 0.0000 | *** |
| CCL25 | TSPO quartile 2 | -0.0115 | 0.3166 | -0.0362 | 0.9712 |  |
| CCL25 | TSPO quartile 3 | 0.5541 | 0.3265 | 1.6972 | 0.0931 |  |
| CCL25 | TSPO quartile 4 | 0.1851 | 0.3407 | 0.5433 | 0.5883 |  |
| CCL25 | Age | 0.0293 | 0.0069 | 4.2555 | 0.0001 | *** |
| CCL25 | Gender (male) | 0.0796 | 0.1691 | 0.4706 | 0.6391 |  |
| CCL25 | Amyloid PET | -0.1740 | 0.1499 | -1.1609 | 0.2488 |  |
| ANGPT1 | (Intercept) | -1.0059 | 0.3837 | -2.6218 | 0.0103 | * |
| ANGPT1 | TSPO quartile 2 | 0.4011 | 0.3796 | 1.0565 | 0.2936 |  |
| ANGPT1 | TSPO quartile 3 | 0.9352 | 0.3914 | 2.3894 | 0.0190 | * |
| ANGPT1 | TSPO quartile 4 | 0.8450 | 0.4085 | 2.0684 | 0.0415 | * |
| ANGPT1 | Age | 0.0040 | 0.0082 | 0.4797 | 0.6326 |  |
| ANGPT1 | Gender (male) | -0.1143 | 0.2028 | -0.5638 | 0.5743 |  |
| ANGPT1 | Amyloid PET | 0.1621 | 0.1797 | 0.9020 | 0.3695 |  |

MODEL 3

| **Protein** | **Term** | **Estimate** | **Std.Error** | **T.value** | **P.value** | **Significance** |
| --- | --- | --- | --- | --- | --- | --- |
| TREM2 | (Intercept) | -2.1687 | 0.8798 | -2.4650 | 0.0156 | * |
| TREM2 | TSPO PET | 1.5901 | 0.9291 | 1.7114 | 0.0904 |  |
| TREM2 | Age | 0.0162 | 0.0062 | 2.6056 | 0.0107 | * |
| TREM2 | Gender (male) | -0.3280 | 0.2024 | -1.6207 | 0.1085 |  |
| TREM2 | Amyloid PET | -0.2922 | 0.1794 | -1.6286 | 0.1069 |  |
| TNFRSF11B | (Intercept) | -2.9170 | 0.8391 | -3.4762 | 0.0008 | *** |
| TNFRSF11B | TSPO PET | 2.0248 | 0.8862 | 2.2848 | 0.0246 | * |
| TNFRSF11B | Age | 0.0180 | 0.0059 | 3.0427 | 0.0031 | ** |
| TNFRSF11B | Gender (male) | -0.2119 | 0.1930 | -1.0979 | 0.2752 |  |
| TNFRSF11B | Amyloid PET | -0.2311 | 0.1711 | -1.3508 | 0.1801 |  |
| IL17RB | (Intercept) | -2.4437 | 0.8855 | -2.7598 | 0.0070 | ** |
| IL17RB | TSPO PET | 1.3924 | 0.9351 | 1.4890 | 0.1399 |  |
| IL17RB | Age | 0.0158 | 0.0063 | 2.5191 | 0.0135 | * |
| IL17RB | Gender (male) | -0.2694 | 0.2037 | -1.3226 | 0.1893 |  |
| IL17RB | Amyloid PET | 0.0139 | 0.1806 | 0.0768 | 0.9389 |  |
| HSPA1A | (Intercept) | -2.5831 | 0.7888 | -3.2748 | 0.0015 | ** |
| HSPA1A | TSPO PET | 0.8076 | 0.8330 | 0.9695 | 0.3348 |  |
| HSPA1A | Age | 0.0239 | 0.0056 | 4.2852 | 0.0000 | *** |
| HSPA1A | Gender (male) | -0.0166 | 0.1814 | -0.0916 | 0.9272 |  |
| HSPA1A | Amyloid PET | 0.1411 | 0.1608 | 0.8770 | 0.3828 |  |
| GAL | (Intercept) | -2.2831 | 0.9094 | -2.5105 | 0.0138 | * |
| GAL | TSPO PET | 1.5352 | 0.9604 | 1.5985 | 0.1134 |  |
| GAL | Age | 0.0131 | 0.0064 | 2.0388 | 0.0444 | * |
| GAL | Gender (male) | -0.0902 | 0.2092 | -0.4310 | 0.6675 |  |
| GAL | Amyloid PET | -0.1255 | 0.1854 | -0.6770 | 0.5001 |  |
| EPCAM | (Intercept) | -1.9821 | 0.8707 | -2.2764 | 0.0252 | * |
| EPCAM | TSPO PET | 1.1556 | 0.9196 | 1.2567 | 0.2121 |  |
| EPCAM | Age | 0.0197 | 0.0062 | 3.2084 | 0.0018 | ** |
| EPCAM | Gender (male) | -0.1474 | 0.2003 | -0.7359 | 0.4637 |  |
| EPCAM | Amyloid PET | -0.2681 | 0.1776 | -1.5097 | 0.1346 |  |
| CXCL1 | (Intercept) | -2.9638 | 0.8030 | -3.6909 | 0.0004 | *** |
| CXCL1 | TSPO PET | 1.8118 | 0.8481 | 2.1364 | 0.0353 | * |
| CXCL1 | Age | 0.0210 | 0.0057 | 3.6934 | 0.0004 | *** |
| CXCL1 | Gender (male) | -0.3307 | 0.1847 | -1.7902 | 0.0767 |  |
| CXCL1 | Amyloid PET | -0.1359 | 0.1637 | -0.8299 | 0.4088 |  |
| CD160 | (Intercept) | -2.5785 | 0.8746 | -2.9481 | 0.0041 | ** |
| CD160 | TSPO PET | 1.3206 | 0.9237 | 1.4297 | 0.1562 |  |
| CD160 | Age | 0.0169 | 0.0062 | 2.7265 | 0.0077 | ** |
| CD160 | Gender (male) | -0.0078 | 0.2012 | -0.0387 | 0.9692 |  |
| CD160 | Amyloid PET | 0.0346 | 0.1783 | 0.1938 | 0.8468 |  |
| CCL25 | (Intercept) | -1.7401 | 0.7507 | -2.3181 | 0.0227 | * |
| CCL25 | TSPO PET | -0.0951 | 0.7928 | -0.1200 | 0.9047 |  |
| CCL25 | Age | 0.0342 | 0.0053 | 6.4480 | 0.0000 | *** |
| CCL25 | Gender (male) | 0.1420 | 0.1727 | 0.8224 | 0.4130 |  |
| CCL25 | Amyloid PET | -0.1778 | 0.1531 | -1.1613 | 0.2485 |  |
| ANGPT1 | (Intercept) | -2.7338 | 0.8874 | -3.0806 | 0.0027 | ** |
| ANGPT1 | TSPO PET | 1.6469 | 0.9372 | 1.7572 | 0.0822 |  |
| ANGPT1 | Age | 0.0111 | 0.0063 | 1.7767 | 0.0790 |  |
| ANGPT1 | Gender (male) | -0.0678 | 0.2041 | -0.3319 | 0.7407 |  |
| ANGPT1 | Amyloid PET | 0.1398 | 0.1810 | 0.7728 | 0.4417 |  |

MODEL 4

| **Protein** | **Term** | **Estimate** | **Std.Error** | **T.value** | **P.value** | **Significance** |
| --- | --- | --- | --- | --- | --- | --- |
| TREM2 | (Intercept) | -0.7412 | 0.3361 | -2.2049 | 0.0300 | * |
| TREM2 | TSPO quartile 2 | 0.2286 | 0.3628 | 0.6301 | 0.5302 |  |
| TREM2 | TSPO quartile 3 | 0.9082 | 0.3800 | 2.3902 | 0.0189 | * |
| TREM2 | TSPO quartile 4 | 0.8563 | 0.3973 | 2.1551 | 0.0338 | * |
| TREM2 | Age | 0.0066 | 0.0076 | 0.8665 | 0.3885 |  |
| TREM2 | Gender (male) | -0.4270 | 0.1973 | -2.1644 | 0.0331 | * |
| TNFRSF11B | Amyloid PET | -1.0467 | 0.3217 | -3.2532 | 0.0016 | ** |
| TNFRSF11B | (Intercept) | 0.2402 | 0.3473 | 0.6917 | 0.4909 |  |
| TNFRSF11B | TSPO quartile 2 | 0.8760 | 0.3637 | 2.4087 | 0.0180 | * |
| TNFRSF11B | TSPO quartile 3 | 0.9132 | 0.3803 | 2.4012 | 0.0184 | * |
| TNFRSF11B | TSPO quartile 4 | 0.0106 | 0.0073 | 1.4476 | 0.1512 |  |
| TNFRSF11B | Age | -0.2838 | 0.1888 | -1.5030 | 0.1363 |  |
| IL17RB | Gender (male) | -0.9791 | 0.3389 | -2.8892 | 0.0048 | ** |
| IL17RB | Amyloid PET | 0.0631 | 0.3658 | 0.1725 | 0.8635 |  |
| IL17RB | (Intercept) | 0.6521 | 0.3831 | 1.7021 | 0.0922 |  |
| IL17RB | TSPO quartile 2 | 0.6266 | 0.4006 | 1.5642 | 0.1212 |  |
| IL17RB | TSPO quartile 3 | 0.0127 | 0.0077 | 1.6457 | 0.1033 |  |
| IL17RB | TSPO quartile 4 | -0.3210 | 0.1989 | -1.6140 | 0.1100 |  |
| HSPA1A | Age | -1.5703 | 0.3048 | -5.1523 | 0.0000 | *** |
| HSPA1A | Gender (male) | 0.0966 | 0.3290 | 0.2935 | 0.7698 |  |
| HSPA1A | Amyloid PET | 0.5717 | 0.3445 | 1.6593 | 0.1005 |  |
| HSPA1A | (Intercept) | 0.5077 | 0.3603 | 1.4092 | 0.1622 |  |
| HSPA1A | TSPO quartile 2 | 0.0213 | 0.0069 | 3.0753 | 0.0028 | ** |
| HSPA1A | TSPO quartile 3 | -0.0516 | 0.1789 | -0.2886 | 0.7736 |  |
| GAL | TSPO quartile 4 | -0.7668 | 0.3432 | -2.2345 | 0.0279 | * |
| GAL | Age | 0.1435 | 0.3704 | 0.3874 | 0.6993 |  |
| GAL | Gender (male) | 0.8841 | 0.3879 | 2.2791 | 0.0250 | * |
| GAL | Amyloid PET | 0.7745 | 0.4057 | 1.9093 | 0.0594 |  |
| GAL | (Intercept) | 0.0063 | 0.0078 | 0.8025 | 0.4243 |  |
| GAL | TSPO quartile 2 | -0.1706 | 0.2014 | -0.8471 | 0.3992 |  |
| EPCAM | TSPO quartile 3 | -0.9586 | 0.3246 | -2.9534 | 0.0040 | ** |
| EPCAM | TSPO quartile 4 | 0.1584 | 0.3503 | 0.4522 | 0.6522 |  |
| EPCAM | Age | 1.0170 | 0.3669 | 2.7718 | 0.0068 | ** |
| EPCAM | Gender (male) | 0.6346 | 0.3837 | 1.6540 | 0.1016 |  |
| EPCAM | Amyloid PET | 0.0100 | 0.0074 | 1.3567 | 0.1782 |  |
| EPCAM | (Intercept) | -0.2340 | 0.1905 | -1.2284 | 0.2225 |  |
| CXCL1 | TSPO quartile 2 | -1.1776 | 0.3013 | -3.9091 | 0.0002 | *** |
| CXCL1 | TSPO quartile 3 | 0.2793 | 0.3252 | 0.8589 | 0.3926 |  |
| CXCL1 | TSPO quartile 4 | 0.9622 | 0.3405 | 2.8254 | 0.0058 | ** |
| CXCL1 | Age | 0.9418 | 0.3561 | 2.6446 | 0.0096 | ** |
| CXCL1 | Gender (male) | 0.0129 | 0.0069 | 1.8838 | 0.0628 |  |
| CXCL1 | Amyloid PET | -0.4088 | 0.1768 | -2.3123 | 0.0230 | * |
| CD160 | (Intercept) | -1.0505 | 0.3284 | -3.1991 | 0.0019 | ** |
| CD160 | TSPO quartile 2 | 0.6608 | 0.3545 | 1.8643 | 0.0655 |  |
| CD160 | TSPO quartile 3 | 1.1929 | 0.3712 | 3.2136 | 0.0018 | ** |
| CD160 | TSPO quartile 4 | 0.8695 | 0.3882 | 2.2399 | 0.0275 | * |
| CD160 | Age | 0.0062 | 0.0075 | 0.8269 | 0.4104 |  |
| CD160 | Gender (male) | -0.0504 | 0.1927 | -0.2616 | 0.7943 |  |
| CCL25 | Amyloid PET | -1.8363 | 0.2876 | -6.3860 | 0.0000 | *** |
| CCL25 | (Intercept) | 0.0932 | 0.3104 | 0.3003 | 0.7646 |  |
| CCL25 | TSPO quartile 2 | 0.6193 | 0.3251 | 1.9051 | 0.0599 |  |
| CCL25 | TSPO quartile 3 | 0.2337 | 0.3399 | 0.6875 | 0.4935 |  |
| CCL25 | TSPO quartile 4 | 0.0259 | 0.0065 | 3.9641 | 0.0001 | *** |
| CCL25 | Age | 0.0505 | 0.1688 | 0.2989 | 0.7657 |  |
| ANGPT1 | Gender (male) | -0.8922 | 0.3427 | -2.6031 | 0.0108 | * |
| ANGPT1 | Amyloid PET | 0.3048 | 0.3700 | 0.8239 | 0.4121 |  |
| ANGPT1 | (Intercept) | 0.8752 | 0.3874 | 2.2589 | 0.0263 | * |
| ANGPT1 | TSPO quartile 2 | 0.8005 | 0.4051 | 1.9758 | 0.0512 |  |
| ANGPT1 | TSPO quartile 3 | 0.0070 | 0.0078 | 0.9027 | 0.3691 |  |
| ANGPT1 | TSPO quartile 4 | -0.0875 | 0.2012 | -0.4349 | 0.6646 |  |

**Supplemental Table 7:** Differentially expressed proteins table for plasma. The table represents the results of the LIMMA model. From the top, to the bottom, top-ranked genes with adjusted p-values (adj.P.Val), log-fold changes (logFC), and associated gene identifiers (UniProt, OlinkID) are displayed. Significant genes were identified using false discovery rate (FDR) adjustment (adjust = "fdr") and prioritized by statistical strength (B statistic). First 50 results are displayed.

| **Protein** | **adj.P.Val** | **P.value** | **t** | **B** | **logFC** | **UniProt** |
| --- | --- | --- | --- | --- | --- | --- |
| C1QA | 0.8339571 | 0.0066301 | 2.7149739 | -3.892867 | 0.5036631 | P02745 |
| ESM1 | 0.8339571 | 0.0068292 | 2.7051577 | -3.940853 | 0.5227301 | Q9NQ30 |
| CHRDL1 | 0.8339571 | 0.0195472 | 2.3349944 | -4.110906 | 0.4343813 | Q9BU40 |
| LAP3 | 0.8339571 | 0.0249752 | 2.2418459 | -4.14169 | 0.4064119 | P28838 |
| TNFAIP8 | 0.8339571 | 0.0245515 | 2.2484482 | -4.146001 | 0.4123815 | O95379 |
| MVK | 0.8339571 | 0.0317378 | 2.147751 | -4.190823 | 0.3899822 | Q03426 |
| FGF19 | 0.8339571 | 0.0311427 | -2.1552985 | -4.194662 | -0.3964098 | O95750 |
| IL1A | 0.8339571 | 0.0337705 | -2.1228533 | -4.227684 | -0.4026666 | P01583 |
| NCLN | 0.8339571 | 0.0360268 | 2.0966744 | -4.234917 | 0.3941502 | Q969V3 |
| DFFA | 0.8339571 | 0.0419189 | 2.0343702 | -4.244817 | 0.3685349 | O00273 |
| VEGFD | 0.8339571 | 0.0419133 | 2.0344258 | -4.252906 | 0.3745299 | O43915 |
| IL13 | 0.8339571 | 0.0440149 | -2.0139937 | -4.25734 | -0.3669961 | P35225 |
| ICA1 | 0.8339571 | 0.0459455 | 1.9959376 | -4.266948 | 0.3646452 | Q05084 |
| CNTNAP2 | 0.8339571 | 0.0458551 | 1.996769 | -4.270379 | 0.3677372 | Q9UHC6 |
| DAPP1 | 0.8339571 | 0.0462424 | 1.9932182 | -4.273898 | 0.3685743 | Q9UN19 |
| DGKZ | 0.8339571 | 0.0494845 | 1.9644355 | -4.280685 | 0.3583818 | Q13574 |
| SIT1 | 0.8339571 | 0.0502705 | 1.9576971 | -4.291218 | 0.363183 | Q9Y3P8 |
| NELL2 | 0.8339571 | 0.0593078 | 1.8859424 | -4.311607 | 0.3406661 | Q99435 |
| CCL21 | 0.8339571 | 0.0603671 | 1.8781407 | -4.329721 | 0.3521669 | O00585 |
| NT5C3A | 0.8339571 | 0.06464 | 1.8477757 | -4.342014 | 0.3462525 | Q9H0P0 |
| AOC1 | 0.8339571 | 0.0664605 | 1.8353406 | -4.342399 | 0.3394879 | P19801 |
| PIK3AP1 | 0.8339571 | 0.0655418 | 1.8415801 | -4.344066 | 0.3446355 | Q6ZUJ8 |
| FABP9 | 0.8339571 | 0.0687165 | -1.820314 | -4.346483 | -0.3346937 | Q0Z7S8 |
| CXCL12 | 0.8339571 | 0.0711482 | 1.8045643 | -4.351182 | 0.3300605 | P48061 |
| FIS1 | 0.8339571 | 0.0733792 | 1.790498 | -4.353005 | 0.3236542 | Q9Y3D6 |
| CCL13 | 0.8339571 | 0.0818715 | 1.7399597 | -4.376901 | 0.3175906 | Q99616 |
| DPP10 | 0.8339571 | 0.0822513 | 1.7378013 | -4.377899 | 0.3173327 | Q8N608 |
| IFNG | 0.8339571 | 0.0810117 | 1.7448771 | -4.37799 | 0.3216683 | P01579 |
| SPINK4 | 0.8339571 | 0.0847726 | -1.7236719 | -4.383086 | -0.3142706 | O60575 |
| CLEC7A | 0.8339571 | 0.0859061 | 1.7174302 | -4.386649 | 0.3142898 | Q9BXN2 |
| ITGA6 | 0.8339571 | 0.090301 | 1.693841 | -4.39417 | 0.3080536 | P23229 |
| IL20RA | 0.8339571 | 0.0913571 | 1.6883107 | -4.401572 | 0.3129629 | Q9UHF4 |
| SIGLEC10 | 0.8339571 | 0.093392 | 1.6777981 | -4.404211 | 0.3094448 | Q96LC7 |
| CKMT1A_CKMT1B | 0.8339571 | 0.0925657 | 1.6820445 | -4.405717 | 0.313862 | P12532 |
| SELPLG | 0.8339571 | 0.0980149 | 1.6545817 | -4.407836 | 0.2990495 | Q14242 |
| FOXO1 | 0.8339571 | 0.098809 | 1.6506824 | -4.413065 | 0.3027563 | Q12778 |
| HLA.E | 0.8339571 | 0.0983412 | 1.6529763 | -4.41438 | 0.3058076 | P13747 |
| TFF2 | 0.8339571 | 0.0971631 | 1.6587925 | -4.415513 | 0.3109067 | Q03403 |
| MGMT | 0.8339571 | 0.0983772 | 1.6527996 | -4.417596 | 0.3096393 | P16455 |
| CXCL1 | 0.8339571 | 0.102388 | 1.6334119 | -4.417723 | 0.2973261 | P09341 |
| CRHBP | 0.8339571 | 0.1026683 | 1.6320797 | -4.418282 | 0.2971506 | P24387 |
| FCRL3 | 0.8339571 | 0.1044031 | 1.6238983 | -4.422639 | 0.2972472 | Q96P31 |
| NME3 | 0.8339571 | 0.1064283 | 1.6144829 | -4.423769 | 0.2925191 | Q13232 |
| IL17A | 0.8339571 | 0.1082402 | -1.6061787 | -4.429263 | -0.2940631 | Q16552 |
| RAB37 | 0.8339571 | 0.1079742 | 1.6073908 | -4.434286 | 0.3015037 | Q96AX2 |
| IL17F | 0.8339571 | 0.1118458 | -1.589976 | -4.434533 | -0.2901903 | Q96PD4 |
| IRAK4 | 0.8339571 | 0.1138192 | 1.5812819 | -4.438652 | 0.2898755 | Q9NWZ3 |
| GZMA | 0.8339571 | 0.1174388 | 1.5656387 | -4.445343 | 0.2884865 | P12544 |
| KLRD1 | 0.8339571 | 0.1194939 | 1.556925 | -4.446565 | 0.2841935 | Q13241 |
| NRTN | 0.8339571 | 0.1239155 | 1.5385677 | -4.455291 | 0.2840826 | Q99748 |
